# NOD1/2 signaling in macrophages drives adaptive immune resistance in cancer

**DOI:** 10.1038/s41392-026-02758-6

**Published:** 2026-07-16

**Authors:** Xiduan Wei, Li Yang, Yuting Wang, Kun Wang, Dan Wang, Mengqian Gao, Xinhua Liu, Xuerui Yang, Suhua Wang, Yiran Zheng, Chunting Wang, Lifang Zhang, Wenjun Yu, Jiawei Wang, Dan Yang, Gang Liu, Yao Ma

**Affiliations:** 1 School of Pharmaceutical Sciences, Tsinghua University, Beijing, PR China; 2 MOE Key Laboratory of Bioinformatics, Center for Synthetic & Systems Biology, School of Life Sciences, Tsinghua University, Beijing, PR China; 3 State Key Laboratory of Holistic Integrative Management of Gastrointestinal Cancers, Beijing Key Laboratory of Carcinogenesis and Translational Research, Department of Hepatobiliary and Pancreatic Surgery Unit I, Peking University, Cancer Hospital & Institute, Beijing, PR China; 4 College of Pharmaceutical Sciences, The Fourth Affiliated Hospital of Soochow University, Soochow Medical School of Soochow University, Soochow University, Suzhou, PR China; 5 Institute of Materia Medica, Chinese Academy of Medical Sciences & Peking Union Medical College, Beijing, PR China; 6 Ningbo Combireg Pharmaceutical Technology Co. Ltd., Ningbo, PR China

**Keywords:** Tumour immunology, Cancer microenvironment, Tumour immunology, Cancer microenvironment

## Abstract

Therapeutic resistance remains a prevalent and intractable clinical challenge across a broad spectrum of human malignancies. Despite extensive investigations, the intricate molecular networks by which the tumor microenvironment (TME) mediates such resistance are not fully understood. In this study, we identified nucleotide-binding oligomerization domain-containing proteins 1 and 2 (NOD1/2) as pivotal regulators of adaptive resistance to diverse antitumor therapies, including immune checkpoint blockade (ICB), adoptive T-cell therapy, and cytotoxic chemotherapy. In murine tumor models, genetic ablation of NOD1/2 or receptor-interacting protein kinase 2 (RIPK2), as well as pharmacological inhibition of RIPK2, remodeled the TME by decreasing immunosuppressive macrophages and boosting CD8⁺ T cell infiltration and cytotoxicity. Mechanistically, NOD1/2 activation in macrophages upregulated programmed death-ligand 1 (PD-L1) expression via the RIPK2/NF-κB signaling axis, establishing an immunosuppressive TME that impaired CD8⁺ T cell-mediated antitumor immunity. Notably, in the clinically relevant setting of immunotherapy resistance, targeted suppression of NOD1/2 signaling in patient-derived peripheral blood mononuclear cells (PBMCs) restored and potentiated ICB responsiveness in patient-derived tumor organoids. Bioinformatic analyses further demonstrated that NOD1/2-associated gene signatures were significantly enriched in tumor-associated macrophages post-therapy. Our findings define NOD1/2 as a novel innate immune checkpoint that orchestrates therapy-induced adaptive resistance and highlight this pathway as a promising target to overcome treatment resistance in refractory cancers.

## Introduction

During the past two decades, cancer has remained the second leading cause of mortality worldwide, accounting for approximately 9.7 million deaths annually.^1^ Despite remarkable advancements in medical science, the global burden of cancer continues to escalate, with an estimated 19.3 million new cases diagnosed in 2020 and projections indicating a 47% increase in incidence by 2040.^2^ This persistent threat underscores the urgent need to unravel the complex molecular mechanisms driving tumor progression and treatment resistance, which constitute major obstacles to achieving long-term remission in cancer patients. Current therapeutic strategies for cancer encompass a broad spectrum of modalities, including surgical resection, radiation therapy, cytotoxic chemotherapy, immunotherapy, oncolytic virotherapy, and emerging gene-editing technologies. Notably, immunotherapies, particularly immune checkpoint blockade (ICB) targeting the PD-1/PD-L1 axis and adoptive T-cell therapies (ACTs), have transformed clinical oncology by demonstrating efficacy against both hematologic malignancies and solid tumors, especially when employed in combination regimens.^3–5^ However, therapeutic efficacy is still constrained by two interrelated mechanisms within the tumor microenvironment (TME): acquired resistance and therapy-induced adaptive resistance (TAR).^6–8^ TAR, in particular, refers to the dynamic remodeling of the TME in response to therapeutic interventions, leading to the emergence of an immunosuppressive niche that blunts antitumor immune responses and compromises treatment outcomes, thus rendering it a critical target for improving cancer therapy.^9^

Nucleotide-binding oligomerization domain-containing proteins 1 and 2 (NOD1 and NOD2) are intracellular pattern recognition receptors (PRRs) belonging to the NOD-like receptor (NLR) family, originally identified as sensors of bacteria-derived γ-D-glutamyl-meso-diaminopimelic acid (iE-DAP) and muramyl dipeptide (MDP), respectively.^10^ Both receptors share a conserved domain architecture, consisting of C-terminal ligand-binding leucine-rich repeats (LRRs), a central nucleotide-binding domain (NOD), and N-terminal caspase recruitment domains (CARDs). Notably, NOD2 uniquely contains dual CARD domains, whereas NOD1 harbors a single CARD motif.^11^ Upon activation, NOD1/2 undergo *S*-palmitoylation, facilitating their membrane localization and interaction with receptor-interacting protein kinase 2 (RIPK2) to initiate nuclear factor kappa B (NF-κB) and mitogen-activated protein kinase (MAPK) signaling cascades that drive proinflammatory responses.^12^ By inducing the production of proinflammatory cytokines and antimicrobial molecules, NOD1/2 coordinate host defense mechanisms, which exert pivotal roles in mediating responses to microbial infection and the pathogenesis of autoimmune/inflammatory disorders, such as Crohn’s disease and rheumatoid arthritis.^13,14^ In recent years, accumulating evidence has revealed that NOD1 and NOD2 exhibit complex, context-dependent functions in cancer pathophysiology, encompassing both oncogenic and tumor-suppressive roles that vary across tumor types. In cervical squamous cell carcinoma, NOD1/2 overexpression is correlated with enhanced tumor cell proliferation, invasion, and migratory capacity.^15^ In colorectal cancer (CRC), agonist-induced activation of NOD1 promotes metastatic progression by increasing cellular adhesion and migration, whereas genetic deficiency of NOD1 has been reported to accelerate estrogen-dependent breast cancer growth.^16,17^ NOD2 suppresses the proliferation of esophageal adenocarcinoma cells via autophagy, yet promotes hepatocellular carcinoma development through a DNA damage-induced autophagy pathway.^18,19^ Additionally, NOD2-overexpressing cells exhibit increased sensitivity to 5-fluorouracil or capecitabine, with suppressed cellular proliferation mediated by modulating the TYMS/PLK1 signaling axis.^20^

Notably, NOD1/2 signaling also displays an analogous functional dichotomy in the crosstalk between the immune system and tumor cells. Activation of NOD1 in tumor-associated macrophages (TAMs) and myeloid-derived suppressor cells (MDSCs) orchestrates CRC tumorigenesis and metastasis via the establishment of immunosuppressive TME.^21,22^ Conversely, studies on hepatocellular carcinoma have demonstrated that NOD1 modulates TAMs to potentiate CD8^+^ T cell-mediated antitumor immunity.^23^ NOD2 also exhibits functional duality in interactions with the gut microbiota to regulate the TME during cancer therapy. For instance, during cyclophosphamide treatment, NOD2 limits the bioactivity of *Enterococcus hirae* and *Bacteroides intestinihominis* to restrict their immunostimulatory effects, including the reduction of regulatory T cells (Tregs) and the promotion of IFN-γ-producing γδT cell infiltration; however, NOD2 senses *Enterococcus* to increase the efficacy of ICB in antitumor immunity.^24,25^ In our prior work, we reported that NOD1/2 antagonists robustly potentiate the antitumor efficacy of paclitaxel (PTX), a phenomenon driven by the targeted modulation of the TME.^26–28^ To translate these preclinical findings into clinical practice, we developed CBRG001 (Salutaxel, Supplementary Table 1), a novel NOD1/2-antagonistic taxane conjugate that exhibits superior antitumor efficacy compared with conventional taxanes and is currently under evaluation in a phase Ib trial (NCT-TR20211264). Although NOD1/2 play multiple biological roles and have therapeutic potential in cancers, their precise molecular mechanisms in TAR remain incompletely elucidated.

In this study, we demonstrated that genetic ablation of both NOD1 and NOD2 markedly improved the therapeutic efficacy of immune checkpoint inhibitors (ICIs). Pharmacological inhibition of NOD1/2 signaling potentiated the antitumor response to multiple clinically relevant therapeutic regimens, including immunotherapy (particularly ICB and ACT) and chemotherapy. Mechanistically, NOD1/2 drives the formation of an immunosuppressive TME and decreases the activation and infiltration density of tumor-infiltrating effector CD8⁺ T cells. This effect is mediated, at least partially, by NOD1/2-dependent upregulation of PD-L1 expression in macrophages via the RIPK2/NF-κB signaling axis. Collectively, our findings reveal a novel mechanism underlying NOD1/2-mediated TAR in the tumor microenvironment, establishing NOD1/2 as a critical regulator of antitumor adaptive immunity and providing a rationale for the clinical application of NOD1/2 inhibitors in combination with existing cancer therapies to overcome treatment resistance.

## Results

### NOD1/2 pathway inhibition synergizes with multimodal cancer therapeutics

**Fig. 1:**
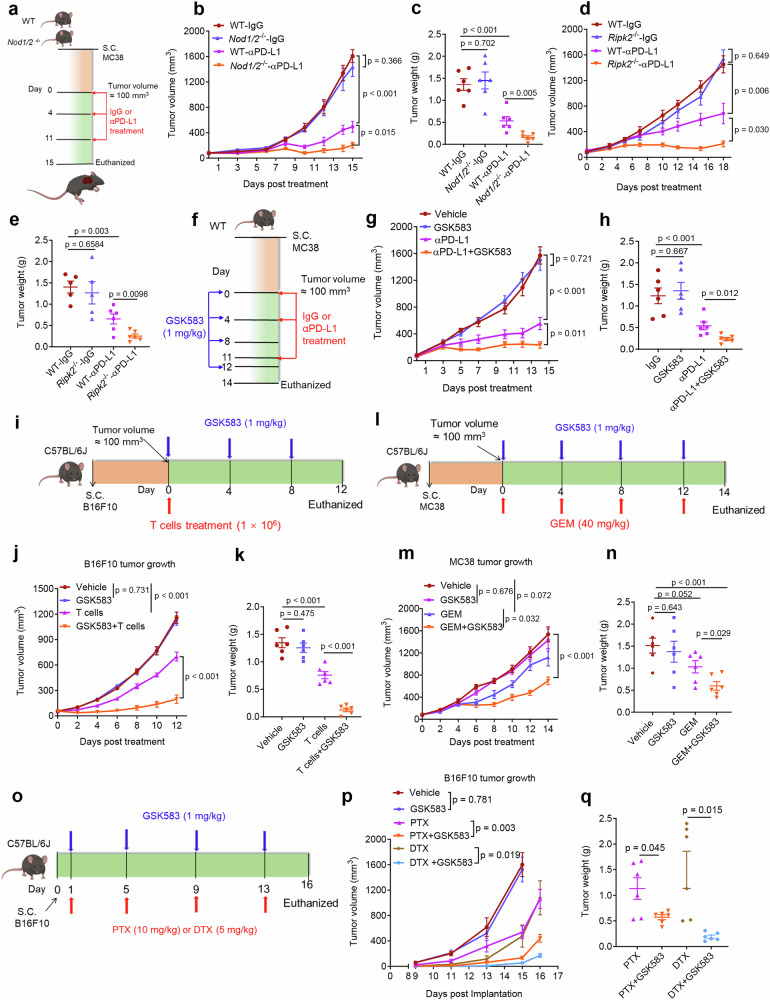
Deficiency or inhibition of NOD1/2 signaling enhances therapeutic responses across multiple cancer treatment modalities. **a** Schematic representation of the MC38 tumor implantation model and treatment timeline for αPD-L1 administration. Wild-type (WT) and *Nod1/2*^−/−^ C57BL/6 J mice were inoculated subcutaneously with 5 × 10⁵ MC38 tumor cells in the right thoracic flank. Once the tumor volume reached approximately 100 mm³, the mice were administered either the IgG isotype control or αPD-L1. **b**, **c** Tumor growth curves and tumor weights of WT and *Nod1/2*^−/−^ mice bearing MC38 tumors following IgG or αPD-L1 treatment (*n* = 6). **d**, **e** Tumor growth curves and tumor weights of WT and *Ripk2*^−/−^ mice bearing MC38 tumors following IgG or αPD-L1 treatment (*n* = 5 or 6). **f** Schematic representation of the tumor model and timeline for combined αPD-L1 and GSK583 treatment. **g**, **h** Tumor growth curves and tumor weights of mice bearing MC38 tumors following αPD-L1 or/and GSK583 treatment (*n* = 6). **i** Schematic representation of the B16F10 tumor implantation model and treatment timeline for ACT with or without GSK583. C57BL/6 J mice were inoculated subcutaneously with 5 × 10⁵ B16F10 tumor cells in the right thoracic flank. Mice were then administered PBS, GSK583, Pmel-1 CD8^+^ T cells (1 × 10⁶ cells), or T cells + GSK583. **j**, **k** Tumor growth curves and tumor weights of mice bearing B16F10 tumors following Pmel-1 CD8⁺ T cell therapy with or without GSK583 treatment (*n* = 6). **l** Schematic representation of the MC38 tumor implantation model and treatment timeline for GEM with or without GSK583. C57BL/6 J mice were inoculated subcutaneously with 5 × 10⁵ MC38 tumor cells in the right thoracic flank. Once the tumor volume reached approximately 100 mm³, the mice were administered GSK583, GEM or GME + GSK583. **m**, **n** Tumor growth curves and tumor weights of mice bearing MC38 tumors following treatment with GEM with or without GSK583 (*n* = 6). **o** Schematic representation of the B16F10 tumor implantation model and treatment timeline for PTX/DTX with or without GSK583. C57BL/6 J mice were inoculated subcutaneously with 5 × 10⁵ B16F10 tumor cells in the right thoracic flank. The day after implantation, the mice were treated with GSK583, PTX, DTX, PTX + GSK583 or DTX + GSK583. **p**, **q** Tumor growth curves and tumor weights of mice bearing B16F10 tumors following PTX or DTX combined with GSK583 treatment (*n* = 6). In all animal experiments, IgG (150 μg per mouse) and αPD-L1 (150 μg per mouse) were administered by intraperitoneal (i.p.) injection on days 0, 4, and 11; GSK583 (1 mg/kg), GEM (40 mg/kg), PTX (10 mg/kg), and DTX (5 mg/kg) were administered every three days by intravenous (i.v.) injection. The data are presented as the mean ± SEM

To evaluate the therapeutic relevance of NOD1/2 signaling in cancer immunotherapy, we first investigated wild-type (WT), *Nod1*-deficient (*Nod1*^−/−^), and *Nod2*-deficient (*Nod2*^−/−^) C57BL/6 J mice in an MC38 colon carcinoma model following αPD-L1 treatment regimens (Supplementary Fig. 1a). No significant differences in tumor growth were observed among the WT, *Nod1*^*−*^^*/*^^*−*^, and *Nod2*^*−*^^*/*^^*−*^ mice treated with the control IgG (Supplementary Fig. 1b–d). Furthermore, αPD-L1 administration induced comparable tumor suppression across all genotypes, as confirmed by equivalent tumor volumes and weights (Supplementary Fig. 1b–d). We subsequently generated *Nod1/2*-double knockout (*Nod1/2*^−/−^) mice by intercrossing single-gene knockout lines and validated the therapeutic response of these mice to αPD-L1 treatment in MC38 tumor-bearing mouse models (Fig. 1a). While tumor progression in the IgG-treated control groups was comparable between the WT and *Nod1/2*^−/−^ mice, αPD-L1 treatment significantly enhanced tumor regression in the *Nod1/2*^−/−^ cohort (Fig. 1b, c). αPD-L1 therapy reduced tumor weight by 89.20% in *Nod1/2*^−/−^ mice compared with a 60.86% reduction in WT controls (Supplementary Fig. 1e). This potentiating effect extended to αPD-1 therapy, with *Nod1/2*^−/−^ mice exhibiting greater reductions in both tumor volume and weight than their WT counterparts did (Supplementary Fig. 1f, g). Since NOD2 has been reported to sense microbiota-derived signals, particularly those from *Enterococcus*, to modulate immunotherapy responses,^25^ we investigated whether intrinsic differences in the gut microbiota between WT and *Nod1/2*^−/−^ mice contributed to the observed phenotypic differences. WT and *Nod1/2*^−/−^mice were cohoused from weaning (3–4 weeks of age) for a minimum of 4 weeks prior to αPD-L1 treatment, a well-established protocol designed to substantially reduce microbiota divergence between genotypes.^29^ The mice remained cohoused until the end of the experiment, and our results revealed that microbiome normalization did not abrogate the enhanced antitumor response to PD-L1 blockade in *Nod1/2*^−/−^ mice, as assessed by tumor volume and weight (Supplementary Fig. 1h, i). A significant difference in the tumor inhibition rate persisted between WT and *Nod1/2*^−/−^ mice following αPD-L1 treatment after they were cohoused (Supplementary Fig. 1j). These data suggest that the enhanced antitumor efficacy of αPD-L1 in *Nod1/2*^−/−^ mice is driven by host genetics rather than a fixed, microbiota-dependent mechanism. Next, we explored the role of receptor-interacting protein kinase 2 (RIPK2), the essential downstream kinase that mediates the NOD1/2 signaling pathway.^30^ *Ripk2*-deficient (*Ripk2*^−/−^) mice exhibited significantly suppressed tumor growth following αPD-L1 therapy (Fig. 1d, e), suggesting that RIPK2 is required for ICI resistance mediated by NOD1/2 signaling. Pharmacological inhibition of RIPK2 with the specific inhibitor GSK583 further validated synergistic tumor suppression when combined with αPD-L1 or αPD-1, which significantly exceeded the efficacy of either monotherapy (Fig. 1f–h, Supplementary Fig. 2a–d). Furthermore, in the B16F10 ICI-resistant tumor model, adjuvant administration of GSK583 also significantly enhanced the antitumor efficacy of αPD-L1 therapy (Supplementary Fig. 2e–g). Taken together, these results indicate that NOD1/2 impairs ICI efficacy via the canonical RIPK2-dependent signaling axis and that genetic or pharmacological disruption of this pathway markedly enhances antitumor responses to PD-1/PD-L1-targeted immunotherapy.

We then assessed whether NOD1/2 inhibition could increase the efficacy of other anticancer therapies. In a well-established ACT model using Pmel-1 CD8^+^ T cells-mediated therapy against subcutaneous B16F10 melanoma,^31^ compared with Pmel-1 CD8⁺ T cell monotherapy, adjuvant administration of GSK583 nearly completely suppressed tumor growth, with 90.62% tumor growth inhibition (Fig. 1i–k, Supplementary Fig. 2h, i). In chemotherapy studies, cotreatment with GSK583 and gemcitabine (GEM) resulted in a 60.46% decrease in tumor weight compared with a 31.94% decrease in tumor weight with GEM monotherapy (Fig. 1l–n, Supplementary Fig. 2j). Consistent synergistic effects were observed in B16F10 models when GSK583 was combined with PTX or docetaxel (DTX) (Fig. 1o–q). Collectively, these findings suggest that activation of the NOD1/2 signaling pathway contributes to TAR across diverse treatment modalities. Targeted inhibition of this pathway significantly enhances tumor sensitivity to both conventional and advanced anticancer therapies, highlighting its potential as a broadly applicable adjuvant therapeutic strategy.

### NOD1/2 signaling orchestrates immunosuppressive reprogramming in tumors undergoing multimodal therapeutic intervention

**Fig. 2:**
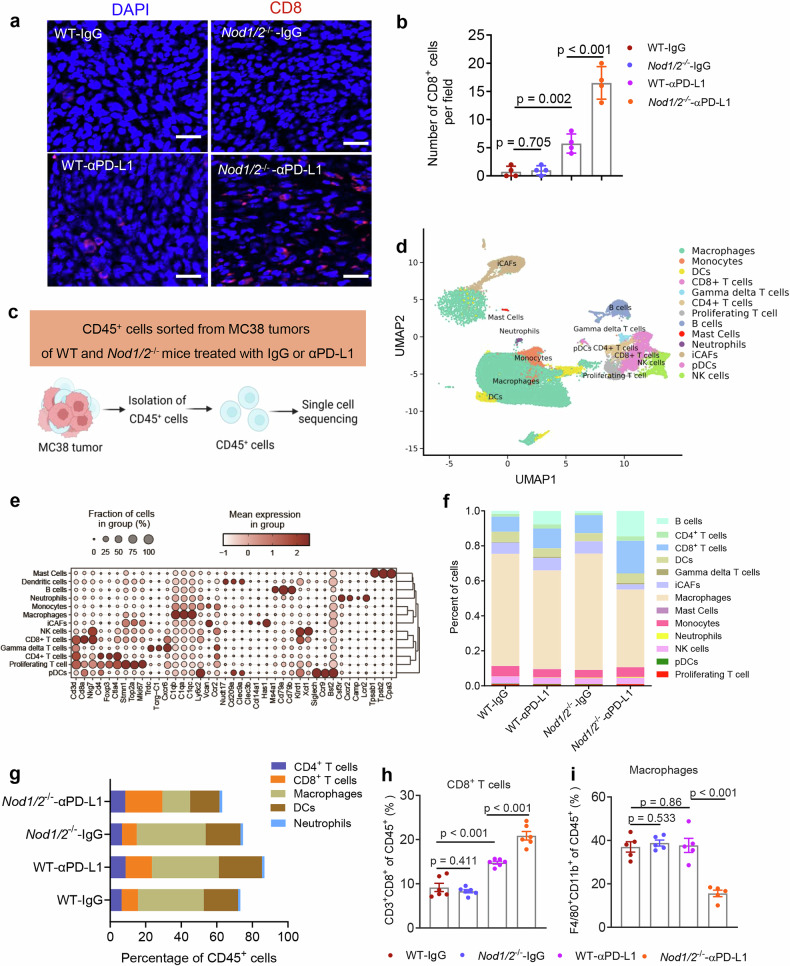
Activation of NOD1/2 signaling impaired tumor immune responses following αPD-L1 treatment. **a**, **b** Representative immunofluorescence images and quantification of CD8⁺ T cells in tumor tissues from WT and *Nod1/2*^−/−^ mice bearing MC38 tumors following IgG or αPD-L1 treatment. CD8, red; DAPI, blue. Scale bar: 50 μm. For each mouse, five representative fields were analyzed (*n* = 4). **c** Schematic of the experimental workflow for scRNA-seq. CD45⁺ cells were sorted from MC38 tumors of WT and NOD1/2-deficient mice treated with IgG or αPD-L1 (*n* = 3, except *Nod1/2*^−/−^ -αPD-L1, where *n* = 2). **d** UMAP plot illustrating distinct immune cell subsets identified from the scRNA-seq data. **e** Bubble plot showing marker gene expression across identified immune cell subsets. **f** Bar plots displaying the relative proportions of immune cell subsets within each group. **g** Flow cytometric analysis of MC38 tumor-infiltrating immune cells in tumor tissues from the four groups (*n* = 5 or 6). Immune subsets were defined as follows: dendritic cells (DCs; CD45⁺CD11c⁺MHCII⁺), macrophages (CD45⁺CD11b⁺F4/80⁺), CD8⁺ T cells (CD45⁺CD3⁺CD8a⁺), and CD4⁺ T cells (CD45⁺CD3⁺CD4⁺). **h**, **i** Quantification of CD8⁺ T cells and macrophages as percentages of total immune cells. The data are presented as the mean ± SEM

Since *Nod1/2* deficiency did not intrinsically suppress tumor growth in our untreated models (Fig. 1b, c, Supplementary Fig. 1f), we investigated whether NOD1/2 signaling contributes to ICI resistance by remodeling the TME. Immunofluorescence analysis revealed that tumors from both WT and *Nod1/2*^−/−^ mice treated with IgG exhibited an immune-desert phenotype, with minimal CD8⁺ T cell infiltration (Fig. 2a, b). αPD-L1 treatment markedly promoted immune infiltration, and the CD8⁺ T cell density in *Nod1/2*^−/−^ tumors was 2.8-fold greater than that in WT tumors (Fig. 2a, b). Moreover, loosened tumor architecture and altered cell density were observed in tumor tissues from the *Nod1/2*^−/−^-αPD-L1 group (Fig. 2a), suggesting that *Nod1/2* deficiency enhanced immune cell infiltration and robust antitumor immune responses following αPD-L1 treatment. Single-cell RNA sequencing (scRNA-seq) of CD45⁺ tumor-infiltrating leukocytes from MC38 tumors was subsequently conducted to systematically characterize NOD1/2-dependent changes in the TME following ICI therapy (Fig. 2c). Dimensionality reduction and clustering revealed 13 distinct immune cell populations, including myeloid cell (macrophage, monocyte, dendritic cell [DC], and neutrophil), lymphoid cell (CD8⁺ T cell, CD4⁺ T cell, γδ T cell, and proliferating T cell), and other cell types (B cell, natural killer [NK] cell, plasmacytoid DC [pDC], mast cell, and inflammatory cancer-associated fibroblast [iCAF]) (Fig. 2d, e, Supplementary Fig. 3a). With respect to the IgG control, *Nod1/2*^−/−^ and WT tumors presented similar immune cell distribution patterns (Fig. 2f, Supplementary Fig. 3b, Supplementary Table 2). In contrast, αPD-L1 treatment markedly altered immune cell infiltration, particularly in *Nod1/2*^−/−^ tumors (Fig. 2f, Supplementary Fig. 3b, Supplementary Table 2). Notably, the frequencies of B cells, CD8⁺ T cells, and neutrophils were markedly elevated, whereas those of CD4⁺ T cells, γδ T cells, NK cells, DCs, pDCs, monocytes, and mast cells were not altered (Fig. 2f, Supplementary Fig. 3b, Supplementary Table 2). Furthermore, the only immune cell populations with reduced infiltration were iCAFs and macrophages, of which macrophages constituted more than 45% of the total immune cell population in each group (Fig. 2f, Supplementary Fig. 3b, Supplementary Table 2).

Given that ICIs mediate their therapeutic effects by activating T cells,^32^ we profiled differentially expressed genes (DEGs) in CD8⁺ T cells isolated from αPD-L1-treated WT and *Nod1/2*^−/−^ tumors. CD8^+^ T cells from αPD-L1-treated *Nod1/2*^−/−^ tumors were significantly enriched in biological processes associated with T cell activation and proliferation signaling pathways (Supplementary Fig. 4). Flow cytometry confirmed increased CD8⁺ T cell infiltration in αPD-L1-treated *Nod1/2*^−/−^ tumors, and further phenotypic characterization of these cells revealed elevated frequencies of IFNγ⁺ and TNFα⁺ CD8⁺ T effector cell subsets in these tumors (Fig. 2g, h, Supplementary Fig. 5a–c). These results indicate that NOD1/2 signaling modulates CD8⁺ T cell infiltration and activation in response to immunotherapy. Furthermore, flow cytometry confirmed a significant reduction in macrophage infiltration in these tumors, with no discernible differences in the abundances of CD4⁺ T cells and DCs (Fig. 2g, i, Supplementary Fig. 5d, e). Although higher neutrophil frequencies were observed in the αPD-L1-treated *Nod1/2*^−/−^ groups than that in the WT-αPD-L1 group by scRNA-seq, no significant differences in these frequencies were detected by flow cytometry (Supplementary Fig. 5f). This observation may be attributable to the low proportion of neutrophils (0.15% and 0.5% in WT-αPD-L1 and *Nod1/2*^−/−^-αPD-L1, respectively; Supplementary Table 2). Moreover, analyses of immunosuppressive cell populations in the TME revealed no significant differences in the frequencies of Tregs, granulocytic MDSCs, or monocytic MDSCs between WT and *Nod1/2*^−/−^ tumors treated with αPD-L1 (Supplementary Fig. 5g–i). Taken together, these results suggest that NOD1/2 signaling primarily modulates the dynamics of macrophages and CD8⁺ T cells during ICI immunotherapy.

We next assessed the tumor immune landscape following pharmacological inhibition of RIPK2. Flow cytometric analysis of MC38 tumors treated with GSK583 in combination with αPD-L1 revealed no significant differences in CD4⁺ T cell frequencies compared with those in tumors treated with αPD-L1 monotherapy, whereas a marked increase in CD8⁺ T cells and their effector subsets (IFNγ⁺ and TNFα⁺ CD8⁺ T effector cells) was observed (Supplementary Fig. 6a–d). Macrophage infiltration was significantly lower in the combination group than in the monotherapy group (Supplementary Fig. 6e). A similar immune modulation pattern was observed in B16F10 tumors treated with GSK583 plus PTX, with consistent alterations in CD8⁺ T cells and macrophage populations compared with those in tumors treated with PTX monotherapy (Supplementary Fig. 6f–h). These results indicate that RIPK2 inhibition alters macrophage and CD8⁺ T cell dynamics across different anticancer regimens. Finally, MTT assays confirmed that GSK583 did not increase the in vitro cytotoxicity of PTX, DTX, or GEM to tumor cells (Supplementary Fig. 7a–c), suggesting that its ability to increase the sensitivity of tumor cells to antineoplastic agents is mediated by immunomodulation rather than direct cytotoxic synergy. Collectively, these findings demonstrate that NOD1/2 signaling reprograms the TME to promote the development of adaptive immune resistance to anticancer therapies.

### NOD1/2 activation drives macrophage-dependent suppression of CD8⁺ T cells

**Fig. 3:**
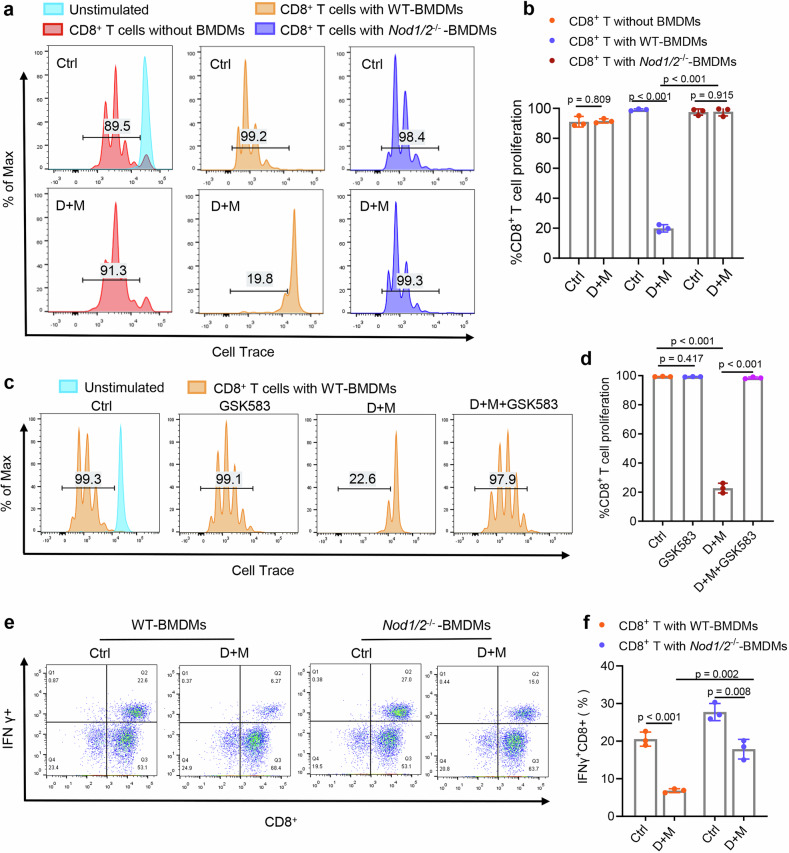
Activation of NOD1/2 signaling promotes immunosuppressive activity in macrophages. **a**, **b** CD8⁺ T cells were isolated from WT mouse splenocytes and labeled with CellTrace Violet. T cell proliferation was assessed by flow cytometry after 3 days of in vitro coculture with BMDMs from WT or *Nod1/2*^−/−^ mice costimulated with C12-iE-DAP and MDP (D + M) (*n* = 3). **c**, **d** Representative histogram and quantification of CD8⁺ T cell proliferation after coculture with BMDMs in the presence or absence of D + M or GSK583 (1.0 μM) (*n* = 3). **e**, **f** CD8⁺ T cells isolated from mouse splenocytes were activated with anti-CD3/anti-CD28 antibodies, IL-2, and IL-12 for 48 h and then cocultured with BMDMs treated with C12-iE-DAP and MDP for 48 h. The proportion of IFNγ⁺ CD8⁺ T cells was determined by flow cytometry (*n* = 3). The data are presented as the mean ± SD

Next, we established a bone marrow-derived macrophage (BMDM)-CD8⁺ T cell coculture system to investigate whether NOD1/2 activation drives an immunosuppressive phenotype in macrophages. Flow cytometric analysis revealed that combined stimulation with C12-iE-DAP (a NOD1-specific agonist) and MDP (a NOD2-specific agonist) resulted in profound suppression of CD8⁺ T cell proliferation in the coculture system of WT-BMDMs with CD8⁺ T cells (99.07% in controls vs. 19.90% in treated groups) but not in the culture system without BMDMs (Fig. 3a, b), suggesting that activation of NOD1/2 specifically suppressed T cell proliferation in a macrophage-dependent manner. This immunosuppressive phenotype was abrogated when CD8⁺ T cells were cocultured with *Nod1/2*^−/−^*-*BMDMs (Fig. 3a, b). Pharmacologically inhibiting RIPK2 with GSK583 similarly reversed the macrophage-mediated suppression of CD8⁺ T cell proliferation, phenocopying the effect of genetic deletion (Fig. 3c, d). Coculture assays of BMDMs with activated CD8⁺ T cells revealed that compared with control macrophages, NOD1/2-activated macrophages markedly reduced the frequency of IFNγ⁺ cytotoxic CD8⁺ T cells to 6.27%, and this inhibitory effect was significantly reversed in the context of NOD1/2 deficiency (Fig. 3e, f). The proportion of IFNγ⁺ CD8⁺ T cells was also lower in *Nod1/2*^−/−^ -BMDMs treated with agonists than in the corresponding controls (Fig. 3e, f). This implies that NOD1/2 ligands may inhibit CD8⁺ T cell activation. Taken together, these results demonstrate that macrophage-intrinsic NOD1/2 signaling impairs CD8⁺ T cell proliferation and activation.

### NOD1/2 activation induces the immunosuppressive functions of macrophages by upregulating PD-L1 expression

**Fig. 4:**
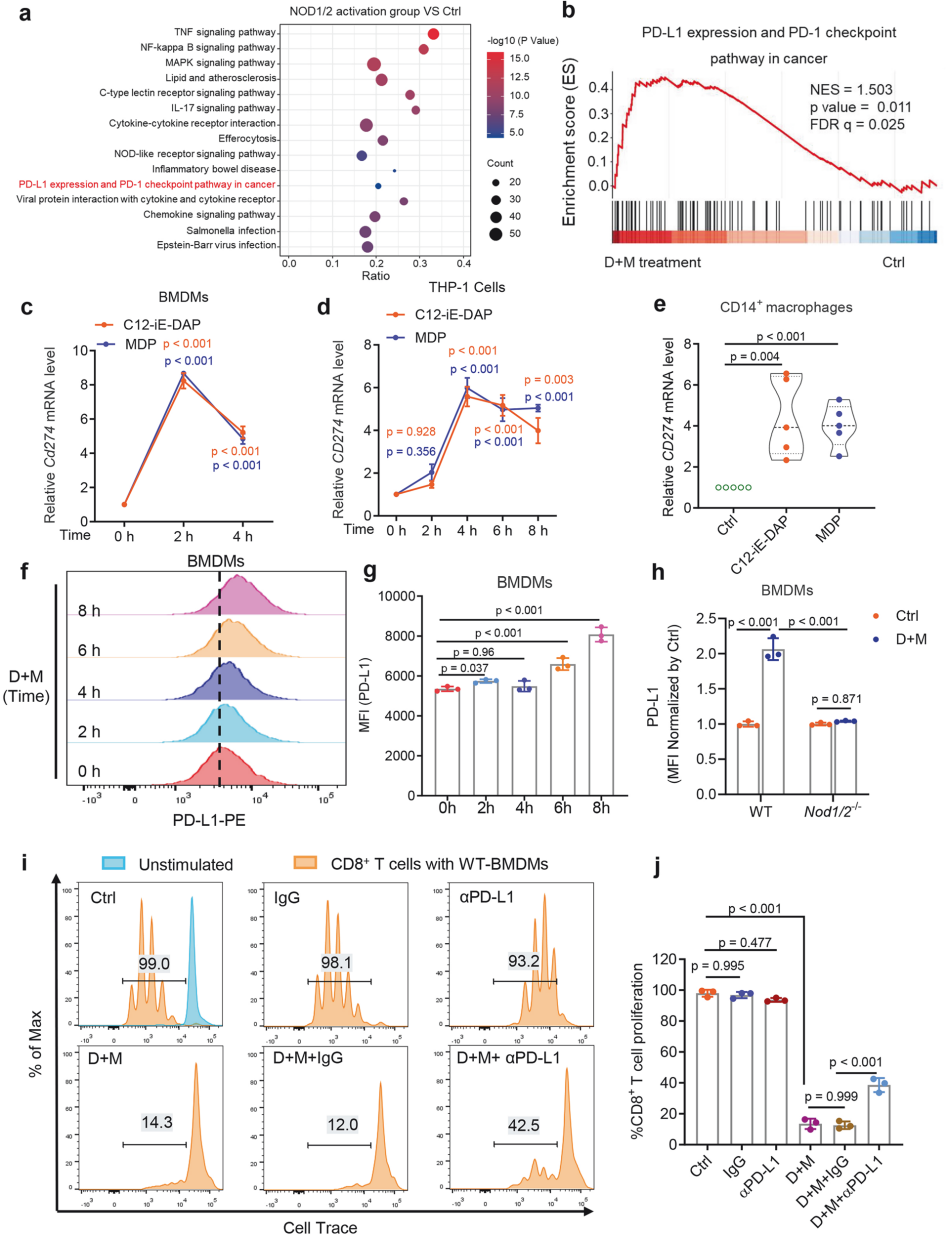
NOD1/2 activation induces the immunosuppressive functions of macrophages by upregulating PD-L1 expression. **a** KEGG pathway enrichment analysis of DEGs in BMDMs costimulated with C12-iE-DAP and MDP (D + M) compared with those in unstimulated controls ( | log₂FC | ≥ 1, *P* < 0.05; *n* = 3). **b** GSEA plots showing a positive association between NOD1/2 activation and PD-L1 expression and PD-1 checkpoint pathway enrichment in cancer. **c**, **d** Relative *CD274* mRNA expression levels in BMDMs and THP-1 cells stimulated with C12-iE-DAP or MDP at the indicated time points (*n* = 3). Statistical analysis was performed relative to the 0 h group. **e** *CD274* mRNA levels in PBMCs after 2 h of stimulation with C12-iE-DAP or MDP (*n* = 5). **f**, **g** Flow cytometry analysis of cell surface PD-L1 levels in BMDMs stimulated with C12-iE-DAP plus MDP at the indicated time points (*n* = 3). **h** Flow cytometry analysis of cell surface PD-L1 levels on WT-BMDMs or *Nod1/2*^−/−^-BMDMs stimulated with C12-iE-DAP plus MDP for 8 h (*n* = 3). **i**, **j** CD8⁺ T cells were isolated from WT mouse splenocytes and labeled with CellTrace Violet. CD8⁺ T cell proliferation was assessed by flow cytometry (*n* = 3). The data are presented as the mean ± SD

To elucidate how NOD1/2 signaling modulates the immunosuppressive function of macrophages, we performed RNA sequencing on BMDMs stimulated with the NOD1/2 agonists C12-iE-DAP and MDP. Enrichment analysis of KEGG pathways for upregulated genes revealed significant enrichment in the PD-L1 and PD-1 checkpoint signaling pathways (Fig. 4a, b). Consistent with this transcriptomic profile, quantitative real-time polymerase chain reaction (qPCR) confirmed that the expression of *CD274* mRNA (encoding PD-L1) was upregulated in both murine BMDMs and the human monocytic and macrophage-like differentiated THP-1 cells following agonist treatment (Fig. 4c, d). This response was also observed in primary CD14⁺ macrophages isolated from human peripheral blood mononuclear cells (PBMCs), where NOD1/2 activation significantly elevated *CD274* expression compared to untreated controls (Fig. 4e). Flow cytometric analysis further validated the NOD1/2-dependent induction of cell surface PD-L1 protein expression, with maximal expression detected at 8 h post-stimulation with NOD1/2 agonist in BMDMs (Fig. 4f, g). Moreover, this agonist-induced PD-L1 upregulation was completely abrogated in *Nod1/2*^−/−^ BMDMs (Fig. 4h), confirming that PD-L1 induction is dependent on NOD1/2 signaling. Functionally, PD-L1 blockade restored the proliferation of CD8⁺ T cells suppressed by NOD1/2-activated macrophages (Fig. 4i, j).

Given that high expression level of PD-L1 on tumor cells is associated with an improved response to ICI,^33^ we examined the baseline expression of *Nod1*, *Nod2*, *Ripk2* and *Cd274* in murine tumor tissues stratified as immunotherapy responders (R) or nonresponders (NR) in TISMO.^34^ No significant differences in these markers were observed across multiple tumor models (Supplementary Fig. 8a–d). Similarly, analysis of cell line with ICI-sensitive (MC38), intermediate (CT26), and ICI-resistant (LLC, 4T1, B16F10) phenotypes revealed no consistent correlations between NOD1/2/RIPK2 and PD-L1 expression at either the transcriptional or protein level (Supplementary Fig. 9a–c). These data suggest that tumor-intrinsic NOD1/2 expression does not affect ICI sensitivity. This conclusion is further supported by our previous finding that NOD1/2 signaling is activated in PBMCs from CRC patients with proficient mismatch repair/microsatellite stable (pMMR/MSS) tumors (Supplementary Fig. 10a), a population with minimal clinical response to ICI.^22^ Furthermore, immunofluorescence staining revealed the colocalization of NOD1 and PD-L1 in TAMs within MSS-CRC tissues (Supplementary Fig. 10b). Taken together, these data demonstrate that NOD1/2 activation drives PD-L1 upregulation and endows macrophages with potent immunosuppressive properties, independent of tumor cell-intrinsic signaling.

### NOD1/2 regulates PD-L1 expression via the RIPK2–NF-κB pathway

**Fig. 5:**
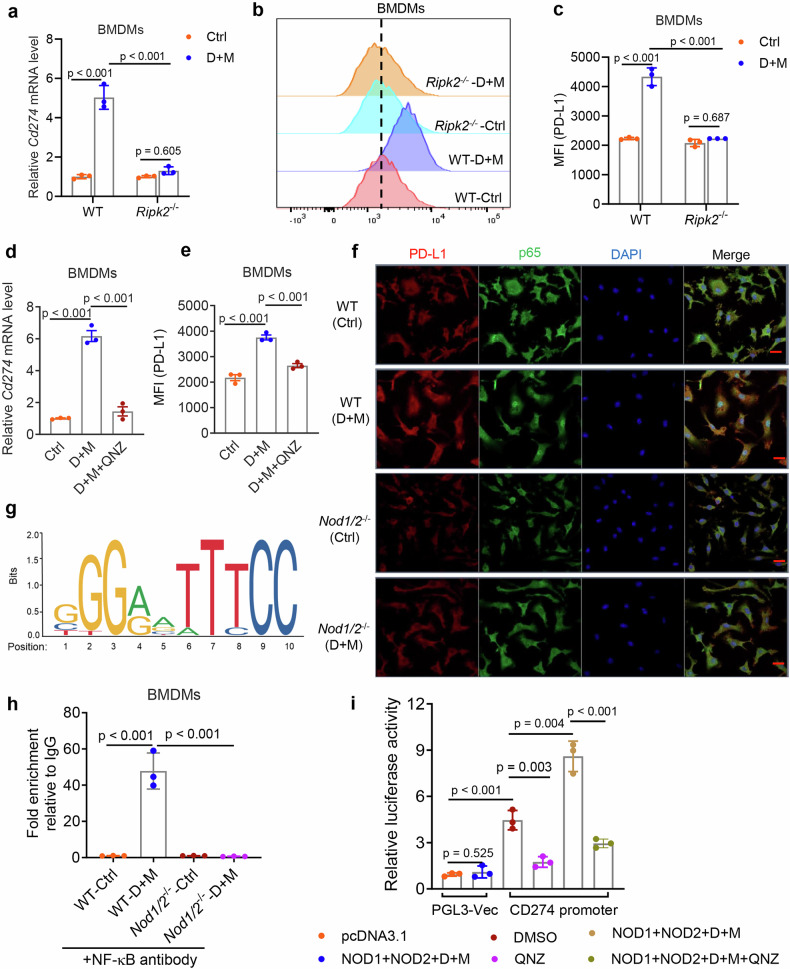
NOD1/2 regulates PD-L1 expression via the RIPK2/NF-κB pathway. **a** Relative *Cd274* mRNA expression levels in BMDMs from WT and *Ripk2*^−/−^ mice; (*n* = 3). **b**, **c** Flow cytometry analysis of cell surface PD-L1 levels on WT-BMDMs or *Ripk2*^−/−^-BMDMs stimulated with C12-iE-DAP plus MDP (D + M) for 8 h (*n* = 3). **d** Relative *Cd274* mRNA expression levels in BMDMs treated with QNZ (an NF-κB inhibitor) for 1 h and then stimulated with C12-iE-DAP and MDP (D + M) for another 2 h (*n* = 3). **e** Flow cytometry analysis of PD-L1 expression on the cell surface of BMDMs treated with QNZ for 1 h and then stimulated with C12-iE-DAP and MDP (D + M) for another 8 h (*n* = 3). **f** Representative fluorescence images of PD-L1 and p65 expression in BMDMs stimulated with C12-iE-DAP and MDP (D + M). PD-L1, red; p65, green; DAPI, blue. **g** The putative NF-κB binding motif in the PD-L1 promoter region, with relative scores determined by JASPAR analysis. **h** ChIP‒qPCR analysis of WT and *Nod1/2*^−/−^ BMDMs treated with C12-iE-DAP and MDP (D + M) for 2 h, showing that activation of *Nod*1/2 promotes the binding of NF-κB to the *Cd274* promoter (*n* = 3). **i** Dual-luciferase reporter assay for measuring PD-L1 promoter activity in HEK-293T cells; the data were normalized to those of the control group (*n* = 3)

To elucidate the underlying signaling mechanism through which NOD1/2 regulates PD-L1, we pharmacologically inhibited NOD1 (ML130) and NOD2 (GSK717) in macrophages stimulated with the NOD1 agonist C12-iE-DAP or the NOD2 agonist MDP. The increase in *CD274* expression induced by each agonist was effectively abrogated by the corresponding specific inhibitor (Supplementary Fig. 11a–b). Moreover, inhibition of RIPK2 with GSK583 significantly blocked *CD274* upregulation in both NOD1/2-activated THP-1 cells and BMDMs (Supplementary Fig. 11a–d). In *Ripk2*-deficient BMDMs, NOD1/2 activation failed to induce PD-L1 upregulation at both the transcriptional and protein levels, as determined by qPCR and flow cytometry (Fig. 5a–c). These results demonstrate that NOD1/2-mediated PD-L1 upregulation is dependent on RIPK2.

NOD1/2 signaling is known to activate multiple downstream cascades, including nuclear factor kappa B (NF-κB), mitogen-activated protein kinase (MAPK), and TANK-binding kinase 1/interferon regulatory factor 5 (TBK1/IRF5) axis.^10^ To identify the key downstream effector that mediates NOD1/2-induced PD-L1 expression, we treated NOD1/2-activated cells with pathway-selective inhibitors. NF-κB inhibition with QNZ markedly reduced *CD274* upregulation in NOD1/2-activated THP-1 cells, whereas inhibition of activator protein 1 (AP-1) with T5224 and TBK1/IKKε with MRT67307 had minimal effects (Supplementary Fig. 11e). This QNZ-mediated suppression of PD-L1 expression was further validated in NOD1/2-activated BMDMs at both the transcriptional and protein levels (Fig. 5d, e). Immunofluorescence staining revealed that the nuclear translocation of p65 (a critical subunit of the NF-κB heterodimer) paralleled the upregulation of PD-L1 expression in NOD1/2-activated BMDMs (Fig. 5f). To further confirm the transcriptional regulation of *CD274* by NF-κB, we predicted *CD274*-targeting transcription factors using the JASPAR database and analyzed publicly available chromatin immunoprecipitation followed by sequencing (ChIP-seq) data GSE131710 from the Gene Expression Omnibus (GEO) database. Both approaches identified NF-κB (RELA) as a putative transcription factor that regulates *CD274* expression (Supplementary Fig. 11f, g). To investigate whether NF-κB directly binds to the *CD274* promoter and modulates its transcriptional activity, we first analyzed the human *CD274* promoter region and identified evolutionarily conserved NF-κB binding motifs (Fig. 5g, Supplementary Fig. 11h). ChIP coupled with quantitative PCR (ChIP‒qPCR) was performed in WT and *Nod1/2*^−/−^-BMDMs, which confirmed the direct binding of NF-κB to the *Cd274* promoter upon NOD1/2 activation (Fig. 5h). A luciferase reporter assay further validated the functional correlation between NOD1/2 activation and increased *CD274* promoter activity (Fig. 5i). Additionally, NF-κB inhibition with QNZ significantly reduced NF-κB occupancy at the *CD27*4 promoter in NOD1/2-activated cells (Fig. 5i). Taken together, these results demonstrate that NOD1/2 activation upregulates PD-L1 expression in a RIPK2/NF-κB-dependent manner.

### Inhibition of NOD1/2 signaling in patient-derived PBMCs potentiated ICB responsiveness in MSS-CRC organoids

**Fig. 6:**
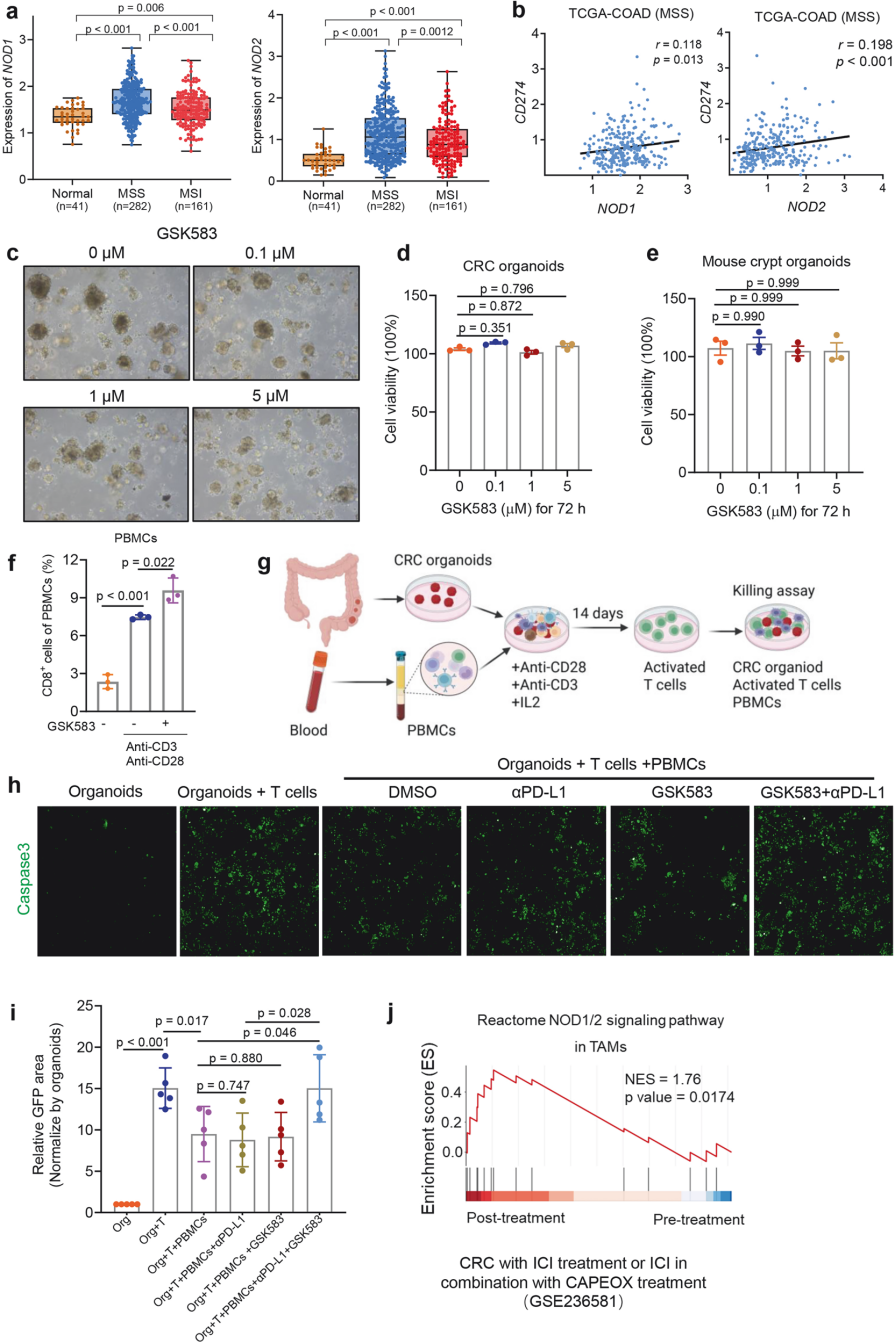
RIPK2 inhibition sensitizes MSS-CRC organoids to immune checkpoint blockade. **a** Expression analysis of *NOD1* and *NOD2* in colon cancer samples from the TCGA; normal tissue (*n* = 41); MSS tumor tissue (*n* = 282); MSI tumor tissue (*n* = 161). **b** Correlation analysis between the expression of *CD274* (encoding PD-L1) and that of *NOD1* or *NOD2* in MSS colon cancer samples from the TCGA cohort (*n* = 282), as assessed using Pearson’s rank correlation. **c** Representative images of CRC organoids treated with GSK583 for 72 h. **d**, **e** The cell viability of CRC organoids and mouse intestinal crypt organoids were detected using a CellTiter-Glo® 3D cell viability assay (*n* = 3). **f** Proportion of CD8⁺ T cells among PBMCs from MSS-CRC patients in the presence or absence of GSK583 (1 μM) (*n* = 3). **g** Schematic of the experimental procedure used to evaluate the cytotoxic activity of tumor-reactive T cells against MSS-CRC organoids. Tumor organoids were derived from MSS-CRC patient samples, and PBMCs were isolated from matched peripheral blood. On the day of coculture, the organoids were dissociated into single cells and plated with PBMCs on anti-CD28-coated plates in the presence of anti-CD3 and IL-2 antibodies for 14 days to generate tumor-reactive T cells. **h**, **i** The cytotoxicity of tumor-reactive T cells to CRC organoids was assessed using a Caspase 3 activity assay kit (*n* = 5). **j** GSEA of NOD1/2 signaling in TAMs from CRC tumor samples following treatment with ICIs (pembrolizumab or sintilimab) or ICIs in combination with CAPEOX compared with the pretreatment state (NES = 1.76, *p* = 0.0174). The data are presented as the mean ± SD

To explore whether targeting the NOD1/2 axis could overcome immunotherapy resistance in a clinically relevant setting, we focused on MSS-CRC, a tumor subtype with minimal responsiveness to ICB, in contrast to mismatch repair-deficient/high microsatellite instability (dMMR/MSI-H) tumors, which frequently exhibit clinical benefit.^35^ Analysis of the Cancer Genome Atlas (TCGA) colon cancer dataset revealed that compared with normal tissues or MSI-high tumors, MSS-CRC tumors exhibited higher expression of both *NOD1* and *NOD2* (Fig. 6a). This difference was particularly significant when MSS tumors were compared to MSI-high (MSI-H) tumors but not to MSI-low (MSI-L) tumors (Supplementary Fig. 12a). Additionally, correlation analysis revealed a positive association between *CD274* expression and that of both *NOD1* and *NOD2* in MSS-CRC samples (Fig. 6b). *CD274* expression was also positively correlated with the expression of *CD68*, a classic marker for macrophage identification (Supplementary Fig. 12b). Collectively, these findings suggest that NOD1/2 signaling may modulate PD-L1 expression in macrophages, thereby promoting ICI resistance in MSS-CRC.

To validate this hypothesis, we established patient-derived MSS-CRC tumor organoids from freshly resected specimens and murine intestinal crypt organoids (Supplementary Fig. 12c, d). CellTiter-Glo assays revealed that the RIPK2 inhibitor GSK583 (0–5 μM) did not exhibit intrinsic cytotoxicity against either human MSS-CRC organoids or murine intestinal crypt organoids (Fig. 6c-e). However, GSK583 significantly increased CD8⁺ T cell proliferation in PBMCs isolated from MSS-CRC patients (Fig. 6f). Based on our earlier finding that the NOD1/2 signaling pathway is activated in PBMCs from MSS-CRC patients^22^ and that GSK583 reverses the macrophage-dependent suppression of T cell proliferation (Fig. 3c, d), these results imply that NOD1/2 signaling within macrophages in PBMCs is activated and contributes to T cell suppression. We further generated tumor-reactive T cells via autologous coculture of PBMCs and MSS-CRC organoids in αCD3/αCD28/IL-2–supplemented medium for 14 days (Fig. 6g), according to previously established protocols.^36,37^ In a subsequent coculture system containing PBMCs, MSS-CRC organoids, and tumor-reactive T cells, the combination of αPD-L1 and GSK583 effectively reversed PBMC-induced immunosuppression and restored tumor-reactive T cell activity against MSS-CRC organoids (Fig. 6g–i). These results demonstrate that inhibition of NOD1/2 signaling synergizes with PD-L1 blockade to reverse immune resistance in MSS-CRC, highlighting a promising combinatorial strategy for this otherwise immunotherapy-refractory population.

### NOD1/2 signaling is activated in cancer patients undergoing therapeutic intervention

To examine the immunological relevance of NOD1/2 signaling in cancer patients undergoing therapeutic intervention, we analyzed scRNA-seq data from the GEO dataset GSE236581,^38^ which includes data from CRC patients treated with neoadjuvant ICI (αPD-1 or αPD-L1) therapy. Analysis of paired tumor biopsies collected before treatment and after the third cycle of ICI monotherapy or combination ICI and CAPEOX therapy revealed significant posttreatment activation of NOD1/2 signaling in TAMs (*n* = 11; normalized enrichment score [NES] = 1.76; *P* = 0.0174) (Fig. 6j). Consistently, upregulation of NOD1/2 signaling in TAMs was observed in liver cancer patients following treatment with αPD-1 or αPD-L1 in combination with anti-CTLA-4 (GSE125449,^39^ *n* = 5; NES = 2.13; *P* < 0.001) (Supplementary Fig. 12e). In patients with triple-negative breast cancer (GSE266919^40^), compared with pretreatment, PTX treatment significantly increased NOD1/2 signaling in TAMs (*n* = 7; NES = 1.65; *P* = 0.031) (Supplementary Fig. 12f). Furthermore, combination therapy with nanoparticle albumin-bound paclitaxel (Nab-PTX) and atezolizumab (ATZ, the PD-L1 antibody) induced robust activation of NOD1/2 signaling in TAMs (*n* = 16; NES = 1.55; *P* = 0.029) (Supplementary Fig. 12g). Together, these findings demonstrate consistent activation of NOD1/2 signaling across multiple tumor types and therapeutic regimens in human patients, corroborating previous evidence from murine models that pharmacological antagonism of this pathway enhances the efficacy of diverse anticancer therapies.

## Discussion

TAR represents a formidable clinical barrier to the effective management of diverse malignancies, with its development driven by a complex interplay of intratumoral heterogeneity, intrinsic molecular aberrations within tumor cells, and dynamic remodeling of the TME.^41–43^ Immune dysfunction and suppression within the TME are well-recognized hallmarks that not only facilitate tumor growth and metastasis but also contribute significantly to the development of therapeutic resistance.^6,44^ Immunotherapeutic strategies designed to reprogram the immunosuppressive TME, whether administered as monotherapies or in combination with other antineoplastic regimens, have revolutionized the landscape of cancer treatment and yielded substantial clinical benefits for patients across multiple tumor types.^45^ Nevertheless, a considerable proportion of patients still exhibit suboptimal responses or develop de novo resistance to currently available immunotherapeutic modalities.^6,46^ In this study, we identified a novel mechanism driving TAR across multimodal oncologic therapies, including ICB, ACT, and cytotoxic chemotherapy. Specifically, this TAR is mediated by the activation of the NOD1/2 signaling pathway in TAMs, which in turn establishes an immunosuppressive TME that impairs CD8⁺ T cell-mediated antitumor immunity.

NOD1 and NOD2 are intracellular PRRs that specifically detect distinct bacterial peptidoglycan fragments: NOD1 recognizes iE-DAP, whereas NOD2 responds to MDP. In the contexts of microbial infection, as well as autoimmune and inflammatory diseases, activation of NOD1/2 confers host protective effects.^10^ In the setting of ICB therapy, our findings reveal functional redundancy between NOD1 and NOD2. Notably, individual deletion of either NOD1 or NOD2 had no impact on the therapeutic response to αPD-L1 treatment. In contrast, simultaneous knockout of both receptors markedly improved tumor sensitivity to αPD-L1 or αPD-1 therapy in murine xenograft models. This phenomenon likely attributed to the fact that NOD1 and NOD2 share a conserved domain architecture and mediate the same downstream signaling, the RIPK2/NF-κB signaling axis in TAMs.^11^ Activation of this axis induces the upregulated expression of PD-L1, thereby establishing an immunosuppressive TME to drive TAR. Individual deletion of either receptor may trigger function compensation, which accounts for the lack of phenotypic changes observed upon single-receptor knockout. This redundancy highlights the importance of targeting both receptors to abrogate their resistance-promoting effects. Consistent with this hypothesis, inhibition of RIPK2, a key downstream kinase that mediates the NOD1/2 signaling,^30^ with the specific inhibitor GSK583 not only potentiated the efficacy of ICB therapy but also increased the sensitivity to ACT and chemotherapy. Collectively, our findings identify NOD1/2 antagonists and RIPK2 inhibitors as promising adjuvants for multiple cancer treatment modalities, providing a novel therapeutic strategy to overcome TAR and improve clinical outcomes for cancer patients.

Although essential for antimicrobial defense, PRRs exhibit functional duality in human pathologies, with the capacity to sense both PAMPs and DAMPs. This unique property positions them as key immunological regulators with paradoxical roles in cancer progression and treatment outcomes.^46^ Recent studies have consistently revealed that NOD1/2 signaling exhibits context-dependent, occasionally contradictory effects and is capable of driving either antitumor immune activation or tumor-permissive immunosuppression.^21–25^ In xenograft models of CRC and melanoma receiving multimodal oncologic therapies, we demonstrated that the sensitizing effect of genetic ablation or pharmacological inhibition of NOD1/2 signaling was mediated primarily by the reversal of macrophage-driven immunosuppression. Analysis of tumor cell lines and baseline tumor tissues revealed no consistent correlation between tumor-intrinsic NOD1/2 expression and ICI sensitivity. Notably, cohousing experiments excluded the possibility that differences in the gut microbiota between WT and *Nod1/2*^−/−^ mice drove the enhanced antitumor sensitivity observed in our experimental system. Additionally, NOD1/2 signaling was found to be activated in TAMs isolated from cancer patients undergoing therapeutic intervention. Further in vitro investigations showed that BMDMs treated with NOD1 or NOD2 ligands acquired immunosuppressive functions, impeding CD8⁺ T cell proliferation and activation. Taken together, these findings indicate that inhibition of NOD1/2 signaling synergizes with multimodal cancer therapeutics by remodeling the TME.

In addition to the dominant alterations in macrophages and CD8⁺ T cells, our scRNA-seq analysis revealed a coordinated reduction in iCAFs and a significant expansion of B cells within the TME of *Nod1/2*^−/−^ mice in response to αPD-L1. Consistent with the findings of previous studies, an increased abundance of tumor-infiltrating B cells and their functional activation are strongly associated with improved ICI response rates and longer patient survival across multiple cancer,^47,48^ whereas the accumulation of iCAFs drives an immunosuppressive TME that promotes acquired resistance during ICI therapy.^49^ The multifaceted shift in the TME of *Nod1/2*^−/−^ mice in response to αPD-L1 suggests that disrupting the NOD1/2 axis facilitates broader TME reprogramming toward a more immune-active state. While our in vitro data establish that macrophage-intrinsic NOD1/2 signaling is sufficient to initiate immunosuppression, the concomitant changes in iCAFs and B cells likely represent complementary features of this successfully redirected microenvironment. Nevertheless, formal in vivo validation using cell type-specific knockout or depletion models will be important for definitively establishing macrophages as the principal cellular compartment driving this adaptive resistance. The functional contribution of these populations, particularly whether the accumulating B cells support antitumor immunity, remains to be determined and presents a compelling direction for future research.

PD-L1 expression on tumor cells is commonly used as a predictive biomarker and companion diagnostic for PD-1/PD-L1 blockade therapy; however, nearly 50% of PD-L1-positive patients fail to respond to anti-PD-1/PD-L1 treatment.^50,51^ Compared with tumor cells, immune cells, such as macrophages and DCs, display significantly elevated PD-L1 expression, and PD-L1-positive immune cells are key mediators of tumor progression.^52–54^ In the present study, we revealed that NOD1/2-mediated upregulation of PD-L1 expression in TAMs enhances the immunosuppressive TME, thereby promoting TAR. Indeed, activation of NOD1/2 in macrophages upregulates PD-L1 expression in a RIPK2/NF-κB-dependent manner. Notably, NOD1 and NOD2 expression was significantly elevated and positively correlated with PD-L1 expression in patients with MSS-CRC who exhibited minimal responsiveness to ICIs.^35^ Furthermore, combining αPD-L1 with a RIPK2 inhibitor effectively overcame hPBMCs-induced immunosuppression and reestablished tumor-reactive T-cell activity against MSS-CRC organoids. These observations reveal that NOD1/2 mediates an immunosuppressive TME by upregulating PD-L1 expression. Notably, we acknowledge that incorporating HLA-matched healthy donor PBMCs as a control in organoid coculture assays (Fig. 6h) would provide a valuable reference for defining tumor-specific immunosuppressive baselines. Future studies incorporating such matched healthy controls will further refine the translational relevance of these findings.

Multiple cytokines and soluble factors, including transforming growth factor-β (TGF-β), interferon-γ (IFN-γ), prostaglandin E₂ (PGE₂), and various interleukins (e.g., IL6, IL-1α, and IL-27), have been shown to regulate PD-L1 expression in TAMs.^55^ Using BMDMs from NOD1/2 double-knockout mice, we confirmed that NOD1/2 signaling is essential for PD-L1 upregulation in macrophages. Mechanistically, NOD1/2 activation engages RIPK2 and subsequently promotes de novo PD-L1 transcription via NF-κB-mediated transcriptional activation. During cancer therapy, DAMPs released by dying or stressed cells serve as upstream stimuli that activate NOD1/2, thereby contributing to the development of TAR. However, the specific DAMP components that initiate NOD1/2 signaling in TAMs remain unidentified and warrant systematic characterization. A key question arising from our findings is how disparate therapeutic modalities-immune checkpoint blockade, cytotoxic chemotherapy, and ACT-affect the activation of the same NOD1/2 signaling axis. We propose that despite their distinct primary mechanisms, these treatments can induce profound immunogenic stress and cell death within the TME, leading to the release of a broad spectrum of DAMPs that act as common upstream triggers. Cytotoxic chemotherapy and ACT are well-established inducers of immunogenic cell death (ICD) and release canonical DAMPs such as ATP and HMGB1. Notably, emerging evidence indicates that ICI therapy, while not directly cytotoxic, can also increase DAMP levels; preclinical studies have shown that short-term anti-CTLA-4 or anti-PD-1 treatment increases the expression of DAMPs, including S100/calgranulin and galectin-3, in tissues and initiates a proinflammatory cascade.^56^ Furthermore, the DAMP landscape is complex and includes potent immunosuppressive molecules such as adenosine, phosphatidylserine, prostaglandin E2 (PGE2), and immunosuppressive extracellular vesicles, which can predominantly suppress antitumor immunity.^57^ Critically, NOD1 and NOD2 are capable of sensing a wide array of such nonbacterial signals, including various DAMPs and cellular stress perturbations. Thus, we propose that the therapeutic stress elicited by multimodal regimens results in a dynamic DAMP milieu, which activates NOD1/2 signaling in TAMs. This in turn drives PD-L1 upregulation, establishing a unified pathway for adaptive resistance across different treatments. Moreover, whether pharmacological NOD1/2 inhibition enhances the efficacy of cytotoxic chemotherapy and ACT through TAM modulation remains to be determined; further mechanistic studies are needed to elucidate these interactions.

The context-dependent function of RIPK2 is highlighted by its contrasting role in pancreatic ductal adenocarcinoma (PDAC), where tumor-intrinsic RIPK2 acts as a constitutive immune checkpoint, the inhibition of which alone suppresses tumor growth and synergizes with anti-PD-1 therapy.^31^ In contrast, in our MC38 and B16 models, this pathway did not intrinsically drive tumor progression but was activated as a TAR mechanism within TAMs. Notably, even though these tumor cell lines exhibited variable baseline levels of Nod1/2 mRNA, this did not correlate with protein abundance or, more importantly, with the functional immunosuppressive outcome in our systems, underscoring that tumor-intrinsic expression is not the primary determinant. This key difference suggests that in our context, the axis operates primarily as a myeloid-centric feedback loop triggered by therapeutic stress rather than as a tumor-autonomous evasion mechanism. Our findings thus reveal a distinct, therapy-responsive role of this pathway and underscore the potential of targeting this therapy-induced adaptive mechanism to overcome treatment resistance across diverse malignancies.

Our study sheds light on the role of NOD1/2 signaling in adaptive resistance to diverse antitumor therapies, but several limitations merit consideration. First, while in vitro and in vivo data strongly implicate macrophages as the primary cellular mediator of NOD1/2-dependent TAR, macrophage-specific NOD1/2 knockout models were not employed; conditional deletion would definitively confirm their cell-autonomous role and exclude contributions from other myeloid or stromal cells. Second, patient-derived organoid studies were limited to MSS-CRC, and whether NOD1/2 inhibition enhances ICB efficacy in other immunotherapy-refractory tumor types (e.g., pancreatic cancer and glioblastoma) remains unclear, highlighting the need to expand studies to additional subtypes for improved generalizability. Third, scRNA‑seq analysis revealed marked expansion of B cells in the TME of *Nod1/2*^*−*^*/*^*−*^ mice treated with αPD‑L1, but the functional role of these B cells remains unresolved and requires further investigation. Finally, the specific DAMPs activating NOD1/2 signaling in TAMs post-therapy remain unidentified; systematic characterization of these DAMPs, such as ATP, HMGB1, or S100 proteins, would clarify upstream TAR triggers and facilitate development of combinatorial strategies targeting both DAMP release and NOD1/2 signaling.

**Fig. 7:**
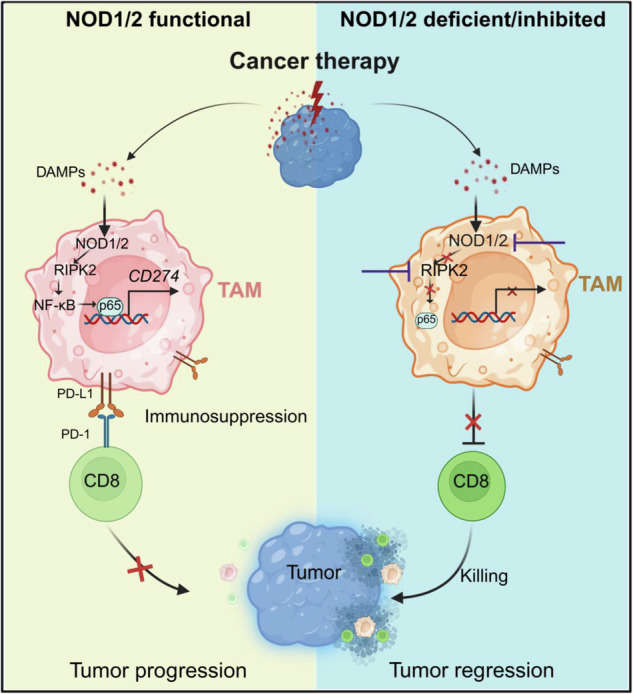
Schematic representation of NOD1/2-mediated immunosuppressive mechanisms in cancer therapy. Pharmacological anticancer treatments promote the release of damage-associated molecular patterns (DAMPs) from injured tumor cells. These DAMPs activate NOD1/2 signaling in tumor-associated macrophages (TAMs), which in turn leads to the upregulation of PD-L1 expression via the RIPK2–NF-κB pathway. This cascade establishes an immunosuppressive tumor microenvironment (TME), thereby driving adaptive resistance and limiting therapeutic efficacy. In contrast, genetic ablation of NOD1/2 or pharmacological inhibition of RIPK2 reprograms the TME, thus enhancing the infiltration and activation of CD8⁺ T lymphocytes. This immunological shift promotes tumor regression and augments the efficacy of anticancer therapies. Schematic diagram generated using BioRender (https://www.biorender.com/)

In summary, genetic deletion or pharmacological inhibition of NOD1/2 signaling sensitizes tumors to multiple therapeutic modalities, including ICB, ACT, and cytotoxic chemotherapy, resulting in partial or complete tumor regression. Functional and mechanistic investigations revealed that NOD1/2 signaling in macrophages promotes an immunosuppressive TME by upregulating PD-L1 expression via the RIPK2–NF-κB axis, thereby impairing effector CD8⁺ T cell activation and infiltration (Fig. 7). Our findings position NOD1/2 as a central checkpoint that governs adaptive immune resistance and highlight the therapeutic potential of combining NOD1/2 inhibition with established anticancer regimens to overcome resistance across diverse malignancies.

## Materials and methods

### Cell lines and cell culture

The murine melanoma cell line B16F10 and the murine fibroblast line L929 were obtained from American Type Culture Collection (ATCC; Manassas, VA, USA). The murine colon adenocarcinoma cell line MC38 was acquired from the China Cell Resource Center of Peking Union Medical College (Beijing, China). The human monocytic cell line THP-1 and human embryonic kidney 293 T (HEK-293T) cells were generously provided by Professor Wanli Liu of Tsinghua University.

MC38, B16F10, and HEK-293T cells were cultured in Dulbecco’s modified Eagle’s medium (DMEM; Invitrogen, Carlsbad, CA, USA) supplemented with 10% (v/v) fetal bovine serum (FBS; Gibco, Australia) and 1% (v/v) penicillin/streptomycin (Invitrogen). L929 cells were maintained in DMEM supplemented with 10% (v/v) FBS and 1 mM sodium pyruvate (Invitrogen) for four consecutive days, after which the L929-conditioned medium was collected. All the cell lines were routinely tested for mycoplasma contamination using a mycoplasma detection kit (M&C Gene Technology, Beijing, China) according to the manufacturer’s protocol.

### Mice

Wild-type (WT) mice and *Nod1*^−/−^, *Nod2*^−/−^, *Nod1/2*^−/−^, *Ripk2*^−/−^, and Pmel-1 transgenic mice (all on the C57BL/6 J strain background) were maintained under specific pathogen-free (SPF) conditions with controlled environmental parameters (12-h light/dark cycle, 22 ± 1 °C ambient temperature, 55 ± 5% relative humidity) and provided ad libitum access to autoclaved food and water at the Animal Research Center of Tsinghua University. WT, *Nod1*^−/−^, and *Ripk2*^−/−^ mice were acquired from Nanjing Biomedical Research Institute of Nanjing University (Nanjing, China). *Nod2*^−/−^ mice and Pmel-1 transgenic mice were obtained from The Jackson Laboratory (Bar Harbor, ME, USA). *Nod1* and *Nod2* double-knockout (*Nod1/2*^−/−^) mice were generated by intercrossing *Nod1*^−/−^ and *Nod2*^−/−^ mice. All experimental protocols were executed in compliance with the ethical standards approved by the Institutional Animal Care and Use Committee (IACUC) of Tsinghua University (Approval No.: 20-LG2).

### Tumor models

For the MC38 colorectal adenocarcinoma model, a suspension containing 5 × 10^5^ MC38 cells in 0.1 mL of phosphate-buffered saline (PBS) was subcutaneously inoculated into the right flank of 6-week-old male mice. Experimental groupings were established through randomized allocation when the tumor volume reached approximately 100 mm³; the mice were randomly divided into different groups and received therapeutic treatment according to predefined protocols. Cohousing studies were performed as previously described.^58^ Age-matched male WT and *Nod1/2*^−/−^ mice aged 3–4 weeks were cohoused in sterilized cages at a ratio of 1:1 (WT: *Nod1/2*^−/−^), with unrestricted access to food and water. No more than 6 mice were housed per cage. Cohoused cohorts received subcutaneous MC38 cell inoculation at 6–7 weeks of age followed by identical therapeutic regimens until the end of the experiment.

In the B16F10 murine melanoma model with αPD-L1 treatment or chemotherapy, male mice (6 weeks old) were subcutaneously implanted with 2 × 10^5^ B16F10 cells in 0.1 mL of PBS. Stratified randomization based on body weight was performed 24 h post inoculation before treatment initiation. For ACT, B16F10 cells were suspended in sterile PBS (5 × 10^6^ cells/mL) and mixed with Matrigel (Corning, NY, USA) at a 9:1 (v/v) ratio. Female C57BL/6 J mice (6–8 weeks) received subcutaneous flank injections of 5 × 10^5^ cells per mouse. Lymphodepletion was achieved via intraperitoneal cyclophosphamide injection (100 mg/kg body weight) administered 7 days post-inoculation. The mice were subsequently randomly divided into different groups, and therapeutic interventions commenced 24 h post-lymphodepletion.

Tumor progression was monitored through tumor volume using the formula: volume (mm³) = 0.5 × major axis × (minor axis) ². The therapeutic efficacy was determined through tumor growth inhibition rate calculation using the formula (V – T)/V × 100%, where V and T represent the final tumor weights from the vehicle control and treatment groups, respectively.

### scRNA-seq analysis

MC38 tumors from WT and *Nod1/2*^−/−^ mice treated with IgG or αPD-L1 were harvested, minced, and enzymatically digested with collagenase IV (0.25%, Sigma‒Aldrich) and DNase I (0.05%, Sigma‒Aldrich) in DMEM/F12 (Invitrogen, Waltham, MA, USA) for 30 min at 37 °C. Single-cell suspensions were generated, and CD45⁺ immune cells were enriched via magnetic bead-based positive selection using a mouse CD45 TIL MicroBead Kit (130-110-618; Miltenyi Biotec, Bergisch Gladbach, Germany) and a Dead Cell Removal Kit (130-090-101; Miltenyi Biotec) according to the manufacturer’s instructions. Briefly, up to 10⁷ cells were resuspended in 90 μL of MACS Buffer (Miltenyi Biotec) per sample, incubated with 10 μL of microbeads for 15 min at 4 °C, and passed through LS columns (130–042–401) mounted on QuadroMACS Separators (Miltenyi Biotec). The columns were subsequently washed, and the remaining CD45⁺ cells were eluted after removal from the magnet.

Next, approximately 1 × 10^4^ CD45⁺ cells per sample were loaded onto the 10x Genomics Chromium platform for single-cell 3′ RNA sequencing. Libraries were prepared using the Chromium Single-Cell 3′ Gene Expression v2 Reagent Kit and sequenced on an Illumina NextSeq system. Raw sequencing data were aligned to the mouse reference genome (mm10-3.0.0) and processed using Cell Ranger (v3.1.0) for barcode demultiplexing, transcript alignment, and gene count matrix generation. Downstream analysis was performed in R (v3.6.1) using Seurat (v3.2.2) and scran (v1.14.6), following best practices outlined in the Bioconductor OSCA workflow (http://bioconductor.org/books/release/OSCA/). Data were normalized, and dimensionality reduction was conducted via principal component analysis (PCA), followed by clustering and visualization using t-distributed stochastic neighbor embedding (t-SNE). Cell populations were annotated on the basis of canonical marker gene expression as follows: macrophages (C1qa, C1qb, C1qc), monocytes (Ly6c2, Vcan, Ccr2), DCs (Nudt17, Cd209a, Clec9a), CD8⁺ T cells (Cd3d, Cd8a, Nkg7), γδ T cells (Cd3d, Trdc, Tcrg-C1, Cxcr6), CD4⁺ T cells (Cd3d, Cd4, Foxp3, Ctla4), proliferating T cells (Stmn1, Top2a, Mki67), B cells (Ms4a1, Cd79a, Cd79b), mast cells (Tpsab1, Tpsb2, Cpa3), neutrophils (Csf3r, Cxcr2, Camp, Lcn2), iCAFs (Col14a1, Has1, Clec3b), pDCs (Siglech, Ccr9, Bst2), and NK cells (Klrd1, Nkg7, Xcl1).

### Flow cytometry

Tumor-infiltrating leukocytes were analyzed as previously described.^59^ In brief, tumor specimens were harvested, mechanically dissociated with surgical scissors, and enzymatically digested in HEPES containing collagenase IV (10 mg/mL; Sigma‒Aldrich), DNase I (200 U/mL; Sigma‒Aldrich), and type V hyaluronidase (1 mg/mL; Sigma‒Aldrich) for 40 min at 37 °C with constant agitation. The cellular suspensions were sequentially filtered through a 70 μm cell strainer, subjected to erythrocyte depletion using RBC Lysis Buffer (BioLegend, San Diego, CA, USA; 5 min incubation), and pretreated with Fc receptor blocking solution (purified anti-mouse CD16/CD32, BioLegend) in cell staining buffer for 30 min. The cells were stained with Fixable Viability Dye eFluor™ 780 (eBioscience; 65-0865-14) as described in the manufacturer’s instructions. Surface staining was carried out by incubation with antibodies against surface markers for 1 h in the dark. The following anti-mouse and cross-reactive antibodies were used: BV650 anti-mouse CD45 (clone 30-F11; 1:200), FITC anti-mouse CD45 (clone 30-F11; 1:400), PE-Cy7 anti-CD11c (clone N418; 1:400), PE anti-CD11b (clone M1/70; 1:200), APC-Cy7 anti-Ly6G (Gr-1) (clone RB6-8C5; 1:200), PerCP-Cy5.5 anti-Ly6C (clone HK1.4; 1:200), BV650 anti-F4/80 (clone BM8; 1:200), APC anti-mouse I-A/I-E (clone M5/114.15.2; 1:1000), FITC anti-mouse CD3 (clone 17A2; 1:200), PE/Cyanine7 anti-mouse CD4 (clone GK1.5; 1:200), and PE anti-mouse CD8a (clone S18018E; 1:400). For intracellular marker (IFNγ and TNFα) staining, the cells were restimulated with Cell Stimulation Mix (eBioscience; 00-4970-93) and incubated for 1 h in a 37 °C/5% CO₂ incubator after surface marker staining. Afterward, Protein Transport Inhibitor Mix (eBioscience; 00-4980-93) was added and further incubated for another 6 h. The cells were subsequently fixed and permeabilized using a Fixation and Permeabilization Kit (eBioscience; 88-8824-00) in accordance with the manufacturer’s instructions and stained with APC/Cyanine7 anti-mouse IFNγ (clone XMG1.2; 1:100) and APC anti-mouse TNFα (clone QA19A26; 1:200). For FOXP3 staining, an eBioscience FOXP3/transcription factor staining buffer kit (Thermo Fisher Scientific, 00-5523-00) was used. The cell stimulation mixture consisted of a mixture of phorbol 12-myristate 13-acetate (PMA) and ionomycin, and the protein transport inhibitor mixture contained brefeldin A and monensin. Flow cytometric analysis was conducted on a BD LSRFortessa (BD Biosciences, San Jose, CA, USA), with data analyzed using FlowJo v10.8.1 software (BD Biosciences).

### In vitro T cell suppression assay

Primary naïve CD8^+^ T cells were isolated from the splenic single-cell suspensions of 6–8-week-old C57BL/6 J mice using a naïve CD8^+^ T cell isolation kit (Miltenyi Biotec) according to the manufacturer’s protocols. Purified cells were labeled with 5 μM CellTrace^TM^ Violet (Thermo Fisher Scientific) for 20 min at 37 °C, followed by washes in RPMI-1640 containing 10% FBS to remove unbound dye. Labeled CD8^+^ T cells were cultured in RPMI-1640 supplemented with 10% (v/v) FBS, 1% (v/v) penicillin/streptomycin, 1X GlutaMAX^TM^ (Invitrogen), 10 mM HEPES (Invitrogen), 1 mM sodium pyruvate (Invitrogen) and 1× 2-mercaptoethanol (Invitrogen) and seeded in round-bottom 96-well plates (1 × 10^5^ cells/well) precoated with 2 μg/mL anti-CD3 (eBioscience; clone 145-2C11). Soluble anti-CD28 antibody (5 μg/mL; clone 37.51; eBioscience) and recombinant IL-2 (10 ng/mL; PeproTech) were added at culture initiation. T-cell suppression was evaluated by coculturing labeled CD8^+^ T cells with BMDMs at a 1:1 ratio for 72 h in a humidified 37 °C/5% CO₂ incubator. Proliferation was quantified via dye dilution using a BD LSRFortessa flow cytometer, and the data were analyzed using FlowJo v10.8.1 software (BD Biosciences).

### In vitro CD8^+^ T cell activation and coculture with BMDMs

Isolated naïve CD8^+^ T cells were activated for 72 h at 37 °C in plates coated with 2 μg/mL anti-CD3 in the presence of anti-CD28 antibody (5 μg/mL), recombinant IL-2 (10 ng/mL), and IL-12 (10 ng/mL; PeproTech) in complete T cell medium. Activated CD8^+^ T cells were collected and cocultured with BMDMs at a 1:1 ratio for 24 h. The cells were stained and analyzed as described in the Mouse Flow Cytometry section.

### Tissue and blood sample collection

Peripheral blood samples were collected from patients with CRC at Peking University Cancer Hospital. Primary tumor tissues were obtained through surgical resection from treatment-naïve patients with microsatellite-stable CRC (MSS-CRC). All study protocols were approved by the Institutional Review Board of Tsinghua University (Protocol No. 20200054). Whole blood samples were processed within 2 h of collection. Peripheral blood mononuclear cells (PBMCs) were purified using Ficoll-Paque Plus density gradient medium (density of 1.077 g/mL; GE Healthcare) following established protocols.^22^ Freshly isolated PBMCs were resuspended at a density of 5 × 10^6^ cells/mL in ice-cold freezing medium, and 1 mL aliquots were immediately transferred to cryovials for cryopreservation using CryoStor CS10 medium (STEMCELL Technologies).

### CRC patient-derived organoid culture

Organoids were established from surgically resected CRC specimens following established protocols with modifications.^36,37^ Fresh tumor tissues were processed within 48 h post-resection, minced into 2–4 mm³ fragments using sterile surgical blades, and sequentially washed in ice-cold DMEM containing 1% FBS and 1% penicillin/streptomycin. Tissue digestion was performed with 2 mg/mL collagenase IV in a 37 °C shaking water bath (100 rpm) for 45–120 min. The digested cellular aggregates were filtered through 100 μm nylon mesh filters (Falcon), pelleted by centrifugation at 500 × *g* for 2 min, and then washed twice with basal culture medium. After the final resuspension in cold Advanced DMEM/F-12 (Thermo Fisher Scientific), the cell matrices were combined with growth factor-reduced Matrigel (Corning; 356231) at a 1:3 (v/v) ratio. The mixture was added to 50 μL of domes in prewarmed 24-well plates and polymerized at 37 °C for 20 min. Organoids were maintained in medium supplemented with Advanced DMEM/F12 (Thermo Scientific) supplemented with 1% penicillin/streptomycin, GlutaMax (Gibco), 10 mM HEPES, B27 (Gibco), 1.25 mM *N*-acetyl-L-cysteine (Sigma), 10 mM nicotinamide (Sigma), 500 ng/mL RSPO1 (Organregen; 861-RS1), 100 ng/mL Noggin (Novoprotein; GMP-CB89), 50 ng/mL Wnt3a (Novoprotein; C22R), 25 ng/mL human EGF (PeproTech; GMP100-15-100), 0.5 μM A83-01 (Selleck; S7692), and 10 μM Y-27632 (Selleck; S1049-10).

### Coculture system of PBMCs, MSS-CRC organoids, and tumor-reactive T cells

Tumor-reactive T cell generation was performed following established protocols.^36^ Briefly, autologous PBMCs and MSS-CRC organoids were cocultured in anti-CD3-precoated (clone OKT3; eBioscience; #16-0037-81) U-bottom 96-well plates (Corning) containing T cell medium supplemented with soluble anti-CD28 (5 μg/mL, clone CD28.2; eBioscience; #16-0289-81) and recombinant human IL-2 (10 ng/mL; PeproTech; #200-02). The coculture system underwent medium replenishment or cellular splitting every 48–72 h. On day 7, activated PBMC populations were transferred to fresh antigen-presenting matrix-coated plates and restimulated with newly prepared CRC organoids. Following 14 days of expansion, tumor-reactive T cells were harvested and cocultured with isolated autologous PBMCs (1:1 ratio) in flat-bottom 96-well plates (Corning) at 5 × 10⁴ cells/well for each population. Concurrently, MSS-CRC organoids underwent apoptosis monitoring through live-cell caspase-3 activation labeling using a GreenNuc™ Assay Kit (Beyotime; #C1168M) before resuspension in T cell medium. A tripartite coculture system was established by introducing labeled organoids into the T cell/PBMC microenvironment. High-content imaging was conducted at the 72-h endpoint using a PerkinElmer Opera Phenix high-throughput confocal system equipped with a 20× water immersion objective. Z-stack acquisition (15 slices/well, 2 μm intervals) enabled comprehensive 3D reconstruction of organoid morphology. Image processing and GFP+ apoptotic area quantification were performed using the Harmony® 4.9 image analysis suite (PerkinElmer), with subsequent morphometric analysis conducted in ImageJ (NIH v1.53) using optimized thresholding parameters.

### Gene expression analysis

Total RNA was extracted from cells using TRIzol reagent (Life Technologies, Carlsbad, CA, USA). Reverse transcription of total RNA (1.0 µg) was performed in a 20 µL reaction volume with a High-Capacity cDNA Reverse Transcription Kit (Life Technologies). Quantitative real-time polymerase chain reaction (qPCR) was performed with SYBR Green Supermix (Life Technologies). The values were relative to those of the housekeeping gene glyceraldehyde-3-phosphate dehydrogenase *(GAPDH)* and were calculated using the 2^–ΔΔCT^ method. The primers used for the qPCR analysis are listed in Supplementary Table 3.

### Chromatin immunoprecipitation (ChIP)-qPCR

ChIP was performed as previously described^60^ with minor modifications. Briefly, BMDMs were fixed with 1% formaldehyde (Sigma‒Aldrich) for 10 min at room temperature, followed by quenching with 125 mM glycine for 5 min. The cells were subsequently lysed in ChIP lysis buffer and sonicated for 5 min (30 s on/off cycles) to shear the chromatin. A representative aliquot of fragmented chromatin was resolved on a 1% agarose gel to verify DNA fragment sizes (200–800 bp) and quantified using a Nanodrop spectrophotometer. For immunoprecipitation, chromatin aliquots were incubated overnight at 4 °C with an anti-NF-κB p65 antibody (Cell Signaling Technology), followed by incubation with protein A/G magnetic beads (Thermo Fisher Scientific) for another 2 h. Immunoprecipitated chromatin complexes were eluted, reverse crosslinked, and purified using phenol–chloroform extraction. Enriched DNA fragments were analyzed by qPCR with primers targeting regions of interest.

### Statistical analysis

Statistical analyses were performed using GraphPad Prism (version 10.0; GraphPad Software). For comparisons between two groups, unpaired Student’s *t* tests were used. For comparisons across more than two groups, one-way analysis of variance (ANOVA) with Tukey’s multiple comparison test was applied. Statistical significance was defined as follows: *P < 0.05, **P < 0.01, and ***P < 0.001. The results are presented as the means ± SDs or means ± SEMs.

All additional materials and methods are detailed in the supplementary information.

## Supplementary information

Supplementary Materials

Raw data

## Data Availability

All the data generated or analyzed during this study are available in the form of source data files. The RNA sequencing and single-cell sequencing data generated in this study have been deposited in the Genome Sequence Archive (Genomics, Proteomics & Bioinformatics 2025) in National Genomics Data Center (Nucleic Acids Res 2025), China National Center for Bioinformation/Beijing Institute of Genomics, Chinese Academy of Sciences (GSA: CRA039635 and CRA039734) that are publicly accessible at https://ngdc.cncb.ac.cn/gsa. The publicly available datasets used in this study are accessible via cBioPortal (http://www.cbioportal.org).
