# Supplementary material for: NOD1/2 signaling in macrophages drives adaptive immune resistance in cancer
Source: Signal Transduct Target Ther. 2026 Jul 16;11:300. doi: 10.1038/s41392-026-02758-6 (PMC13385828; doi:10.1038/s41392-026-02758-6)
Supplement: Supplementary file 1 — Supplementary Materials [file 41392_2026_2758_MOESM1_ESM.docx]

**Supplementary Materials for**

**NOD1/2 signaling in macrophages drives adaptive immune resistance in cancer**

Xiduan Wei^1,#^, Li Yang^3,#^, Yuting Wang^1,#^, Kun Wang^4,#^, Dan Wang^1^, Mengqian Gao^5^, Xinhua Liu^1^, Xuerui Yang^3^, Suhua Wang^2^, Yiran Zheng^5^, Chunting Wang^1^, Lifang Zhang^6^, Wenjun Yu^6^, Jiawei Wang^1^, Dan Yang^1^, Gang Liu^1,*^ Yao Ma^2,*^

Correspondence to: **mayao@imm.ac.cn**

**This PDF file includes:**

Materials and Methods

Supplementary Text

Figures S1 to S12

Tables S1 to S3

Materials and Methods

***Adoptive T-cell*** ***therapy (ACT) in animals***

T cell isolation and activation: splenic single-cell suspensions were prepared from 6-8-week-old Pmel-1 transgenic mice by mechanical dissociation and then filtrated using 70 μm cell strainers followed by centrifugation (700 × *g*, 4 min). Erythrocyte depletion was performed using ACK lysis buffer (MilliporeSigma, Burlington, MA, USA). Purified splenocytes were resuspended in lymphocyte culture medium (LCM) containing gp100_25-33_ peptide (5 μg/mL; R&D Systems, Minneapolis, MN, USA), murine IL-2 (10 ng/mL; R&D Systems), and murine IL-7 (1 ng/mL; R&D Systems). LCM consisted of complete RPMI 1640 medium supplemented with 10% FBS, 100 U/mL penicillin, 0.1 mg/mL streptomycin, 10 mM HEPES (Invitrogen), 1 mM sodium pyruvate, and 1% (v/v) MEM non-essential amino acids (Invitrogen). Following 72-hour activation at 37°C, lymphocytes were density-separated using Ficoll-Paque Plus (1.077 g/mL; GE Healthcare, Chicago, USA) and subsequently expanded in cytokine-enriched LCM (IL-2/IL-7, 10 ng/mL each) for 48 h prior to adoptive transfer.

***Tissue processing and immunofluorescence***

Primary tumor tissues from CRC-LM patients and tumor tissues from WT and *Nod1/2^⁻/⁻^* mice bearing MC38 tumors following IgG or αPD-L1 treatment were dissected and embedded in Tissue-Tek O.C.T. 5-μm-thick O.C.T. tissue cryosections were stained with primary antibodies and incubated with Alexa Fluor 488 or 647 secondary antibodies. DAPI was used for nuclear counterstaining, and fluorescent images were photographed using a confocal microscope (LSM980 Airyscan2, Zeiss) and analyzed with Imaris 7. Antibodies used were anti-NOD1 antibody (abcam; ab189409; 1:100), anti-PD-L1 antibody (eBioscience; 14-5982-82; 1:100), anti-CD16 antibody (abcam; ab308605), anti-CD8 antibody (eBioscience; 14-0081-82; 1:200). To ensure strict comparability across all groups for analyzed CD8, field-of-view (FOV) images were captured from the tumor core region (excluding the tumor-stroma edge) of each sample using a confocal microscope with a 20× objective lens; expanded FOV images were acquired via ZEN3.1 (Blue) software with a unified fixed rectangular ROI size (W = 2000, H = 2000). For CD8⁺ cell quantification in Fig. 2B, five representative, non-overlapping FOVs were selected per tumor sample for cell counting, with the average value calculated for each sample; a total of four biological samples were analyzed for each experimental group.

***Human PBMCs derived macrophages***

Human PBMCs were isolated from the buffy coats of anticoagulated blood from healthy blood donors (Beijing Red Cross blood center, China) by gradient centrifugation on Lymphoprep (Axis-Shield), and monocytes were further purified by positive magnetic separation of CD14^+^ cells (MiltenyiBiotec). Macrophages in PBMCs were derived from CD14^+^ blood monocytes by culturing in DMEM medium (Gibco) supplemented with 10% FBS (Gibco) and 20 ng/mL of M-CSF (PeproTech, Rocky Hill, NJ, USA) for 3 days, then supplemented with 20 mL fresh medium for another 4 days.

***Plasmid construction***

pGL3-enhancer luciferase reporter and pRL-TK Renilla luciferase plasmids were purchased from YouBio and were confirmed by sequencing. NOD1 and NOD2 overexpression plasmids were purchased from Invivogen. Transfections were performed using Lipofectamine 2000 reagent (Invitrogen) according to the manufacturer’s instructions.

***Dual-luciferase reporter assay***

Firstly, 293T cells were seeded into 24-well plates. Twelve hours later, cells were co-transfected with luciferase expression constructs and Renilla luciferase plasmids using Lipofectamine 2000. Twenty-four hours later, luciferase activities following indicated treatments were measured using a Dual Luciferase Reporter Assay Kit (Vazyme, Nanjing, China) with a luminometer according to the manufacturer’s instructions. The firefly luciferase values were normalized to Renilla values.

***Macrophage differentiation***

THP-1 monocytes were differentiated with 50 ng/mL phorbol 12-myristate 13-acetate (PMA) (InvivoGen, San Diego, CA, USA) for 48 h, followed by incubation in complete medium for another 24 h to rest. Cells were cultured in RPMI-1640 supplemented with 1% (v/v) FBS overnight for starvation before treatment with C12-IE-DAP (InvivoGen) or MDP (InvivoGen).

For generation of bone-marrow-derived macrophages (BMDMs), bone marrow was isolated from C57BL/6J mice (6-to 8-week-old) femurs and tibias, followed by centrifugation at 300 × *g* for 5 min to obtain bone marrow cells and then cultured for 7 days in DMEM supplemented with 20% (v/v) FBS and 20% (v/v) L929 cell culture supernatant. After 7 days, supernatant and non-adherent cells were removed by washing with PBS, and adherent cells were harvested by incubation in ice-cold PBS for 10 min before gentle pipetting.

***Isolation of mouse crypt and organoid cultures***

Fresh proximal intestinal segments were dissected and longitudinally incised in ice-cold PBS. Following villus removal through gentle scraping with a surgical blade, tissues were minced into 2-5 mm^3^ fragments and subjected to 5 mM EDTA incubation for 30 min at 4°C with periodic agitation. The resulting cell suspension was mechanically dissociated through a 70-μm cell strainer (Corning) to isolate intact crypt structures. Crypt-enriched fractions were pelleted by centrifugation at 300 × *g* for 3 min and subsequently washed twice with complete culture medium (bioGenous; K2001-MI). For organoid establishment, purified crypts were resuspended in EasyGel matrix (bioGenous) at a density of 50 μL per well in 24-well plates. Following polymerization of the EasyGel matrix (37°C, 20 min), 500 μL of complete culture medium was carefully overlaid. Organoid cultures were maintained under standard conditions (5% CO₂, 37°C humidified atmosphere) with medium replenishment every 2 days.

***Cell viability assay for organoids***

CRC-organoids and murine intestinal crypt organoids were enzymatically dissociated using TrypLE Express solution (Gibco; 12605028) through a 10-minute incubation at 37°C combined with mechanical trituration. The cell clusters (2-5 cells/cluster) were pelleted by centrifugation at 300 × *g* for 3 min, followed by two washes with complete culture medium. The cellular pellet was reconstituted in a 1:2 (v/v) matrix mixture of culture medium and EasyGel (bioGenous) with 10 μL aliquots seeded into 96-well flat-bottom plates (Corning). Following matrix polymerization (20 min, 37°C), 100 μL of maintenance medium was overlaid. After 24 h stabilization, experimental wells received GSK583 treatment at specified concentrations. Following 72-h pharmacological exposure, morphological analysis of organoids was performed using bright-field microscopy (Nikon Eclipse Ti) and viability assessment was conducted by adding 100 μL CellTiter-Glo 3D Reagent (Promega; G9682) per well and detected according to the manufacturer’s protocol.

***Gene set enrichment analysis (GSEA)***

Three independent scRNA-seq datasets of human tumor tissues were retrieved from the GEO database: GSE236581 (colorectal cancer), GSE266919 (triple-negative breast cancer, TNBC), and GSE125449 (intrahepatic cholangiocarcinoma). These datasets included transcriptomic profiles of tumor samples collected before and after specific treatment regimens. Macrophages were identified based on expression of C1QA, C1QB, and C1QC markers. Differentially expressed genes, defined by |log2 fold change| ≥ 1 with adjusted *p*-value < 0.05, were analyzed using the limma package in R. Genes analyzed in the NOD1/2 signaling pathway were obtained from public databases,^1^ including *AAMP*, *BIRC2*, *BIRC3*, *CARD9*, *CASP1*, *CASP2*, *CASP4*, *CASP8*, *CASP9*, *CHUK*, *CYLD*, *IKBKB*, *IKBKG*, *IRAK1*, *IRAK2*, *ITCH*, *MAP2K6*, *MAP3K7*, *MAPK11*, *MAPK12*, *MAPK13*, *MAPK14*, *NOD1*, *NOD2*, *RIPK2*, *RPS27A*, *TAB1*, *TAB2*, *TAB3*, *TNFAIP3*, *TRAF6*, *UBA52*, *UBB*, *UBC*, *UBE2N*, and *UBE2V1*. GSEA results were visualized using the ggplot2 and ClusterProfiler packages in R. The minimum and maximum gene set sizes were set to 10 and 1000, respectively. *P*-value < 0.05 was considered significantly enriched.

**Reference**

1 Ruiz-Lafuente, N., Muro, M., Minguela, A. & Parrado, A. The transcriptional response of mouse spleen B cells to IL-4: Comparison to the response of human peripheral blood B cells. *Biochemistry and Biophysics Reports* **16**, 56-61 (2018).

Supplementary Figures and Figure legends

**
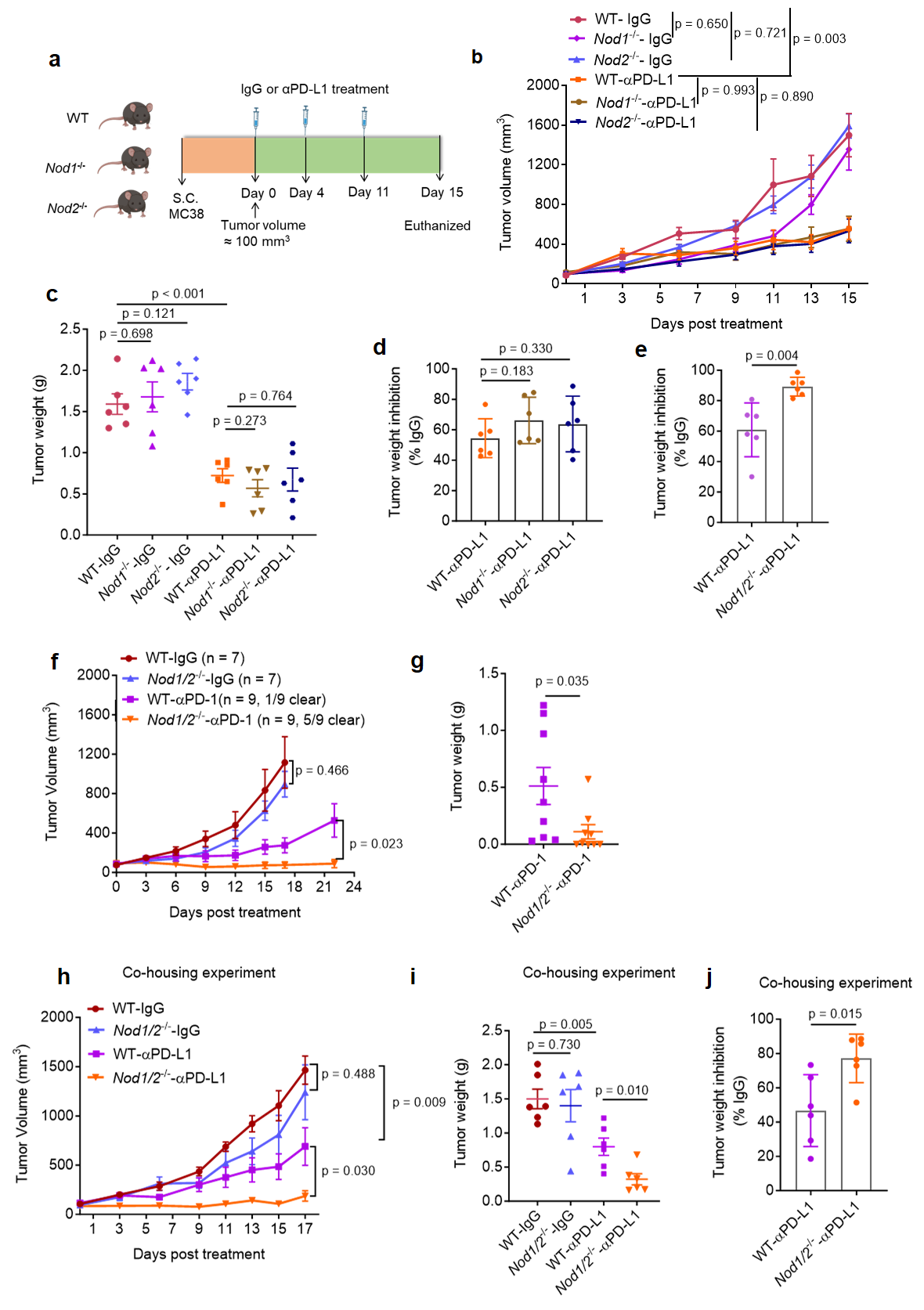
**

**Figure S1. Genetic deletion of NOD1/2** **enhances tumor sensitivity to ICI therapy. a** Schematic of MC38 tumor growth model and the timeline with αPD-L1 treatment in WT, *Nod1*^-/-^, or *Nod2*^-/-^ C57BL/6J mice. Mice were subcutaneously inoculated with 5 × 10^5^ MC38 cells in 100 μL PBS on the right thoracic flank. When tumor volumes reached approximately 100 mm^3^, mice were treated with either an IgG isotype control or αPD-L1. **b-d** Tumor growth curves, final tumor weights and tumor weight inhibition rate in WT, *Nod1*^-/-^, or *Nod2*^-/-^ mice bearing MC38 tumors following administration of IgG or αPD-L1 (*n* = 6). The inhibition rate of αPD-L1 was calculated relative to the corresponding IgG-treated control group. **e** Tumor weight inhibition rate of αPD-L1 treatment in WT or *Nod1/2*^-/-^ mice calculated relative to the corresponding IgG-treated control group (*n* = 6). **f, g** Tumor growth curves and final tumor weight in mice bearing MC38 tumors following αPD-1 treatment (*n* = 7 to 9 per group). **h-j** 4-week-old male WT and *Nod1/2*^-/-^ mice were cohoused in sterile cages at a 1:1 ratio (WT: *Nod1/2*^-/-^) and received subcutaneous MC38 cell inoculation at 6 weeks of age followed by IgG or αPD-L1 treatment. Tumor growth curves, final tumor weight, and tumor weight inhibition rate in mice bearing MC38 tumors following αPD-L1 treatment (*n* = 6). The inhibition rate of αPD-L1 was calculated relative to the corresponding IgG-treated control group. In all animal experiments, IgG (150 μg per mouse), αPD-L1 (150 μg per mouse) and αPD-1 (150 μg per mouse) were administered by intraperitoneal (i.p.) injection on days 0, 4, and 11. The data are presented as the mean ± SEM. Statistical significance was determined using Student’s *t*-test.


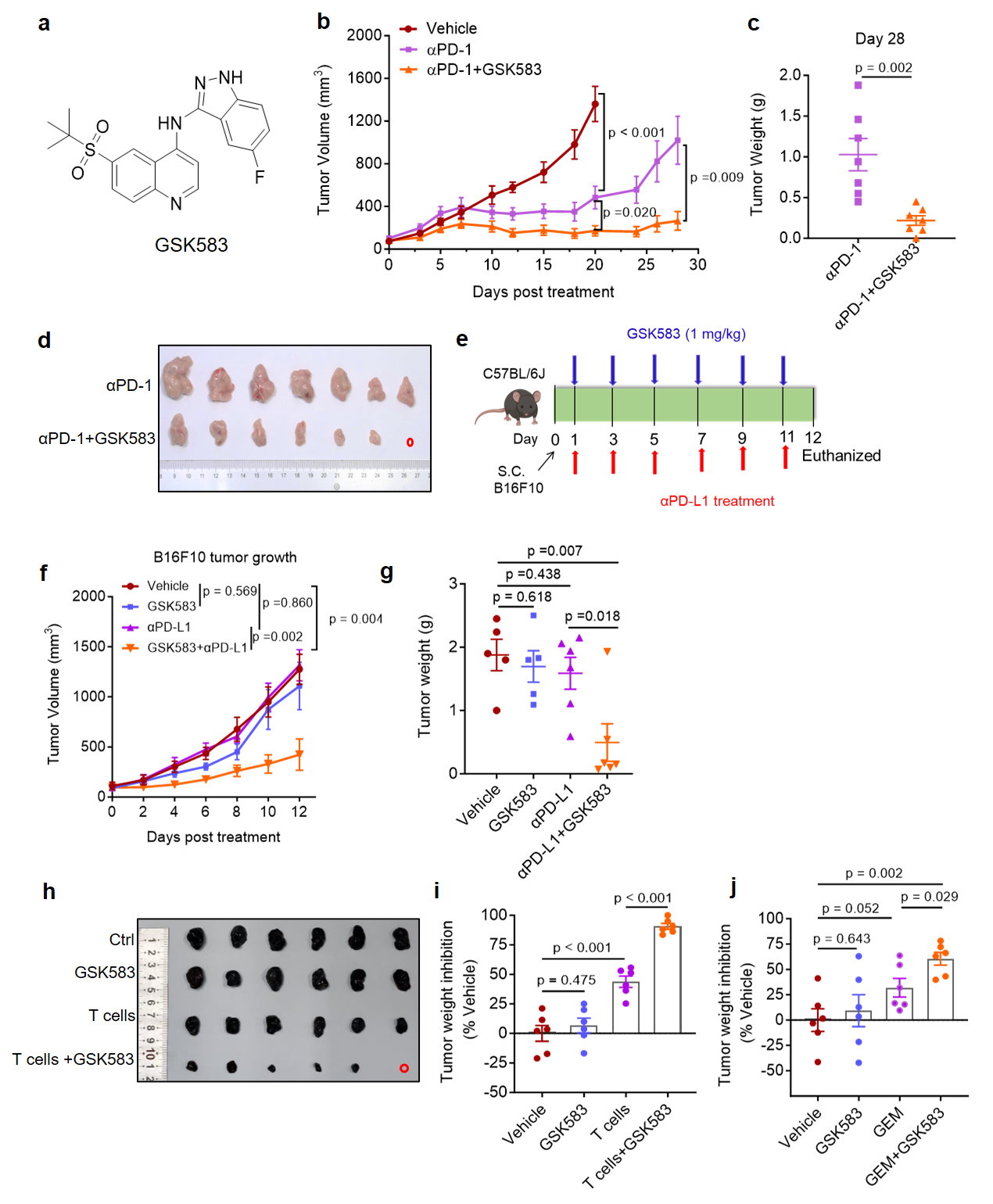


**Figure S2. Inhibition of RIPK2 improves therapeutic responses across multiple cancer treatment modalities.** **a-d** The chemical structure of GSK583. Tumor growth curves, final tumor weight, and photograph of final tumor on day 28 of MC38 tumors following αPD-1 and/or GSK583 treatment. IgG (150 μg per mouse) and αPD-1 (150 μg per mouse) were administered by i.p. injection on days 0, 4, and 11; GSK583 (1 mg/kg) was administered every three days by intravenous injection (i.v.) (n = 6 or 7 per group). **e** Schematic representation of the B16F10 tumor implantation model and timeline for combined αPD-L1 and GSK583 treatment. IgG (150 μg per mouse) and αPD-L1 (150 μg per mouse) were administered by i.p. injection every other day. GSK583 (1 mg/kg) was administered every other day by i.v.. **f, g** Tumor growth curves and final tumor weight of B16F10 tumor following αPD-L1 or/and GSK583 treatment (*n* = 5 or 6 per group). **h** Photograph on day 12 of B16F10 tumor following CD8^+^ T cells and/or GSK583 treatment. **i** Tumor weight inhibition rate of CD8^+^ T cells and/or GSK583 treatment in B16F10 tumors, calculated relative to the Vehicle control group (*n* = 6). **j** Tumor weight inhibition rate of GEM and/or GSK583 treatment in MC38 tumors, calculated relative to the Vehicle control group (*n* = 6). The data are presented as the mean ± SEM. Statistical significance was determined using Student’s *t*-test.


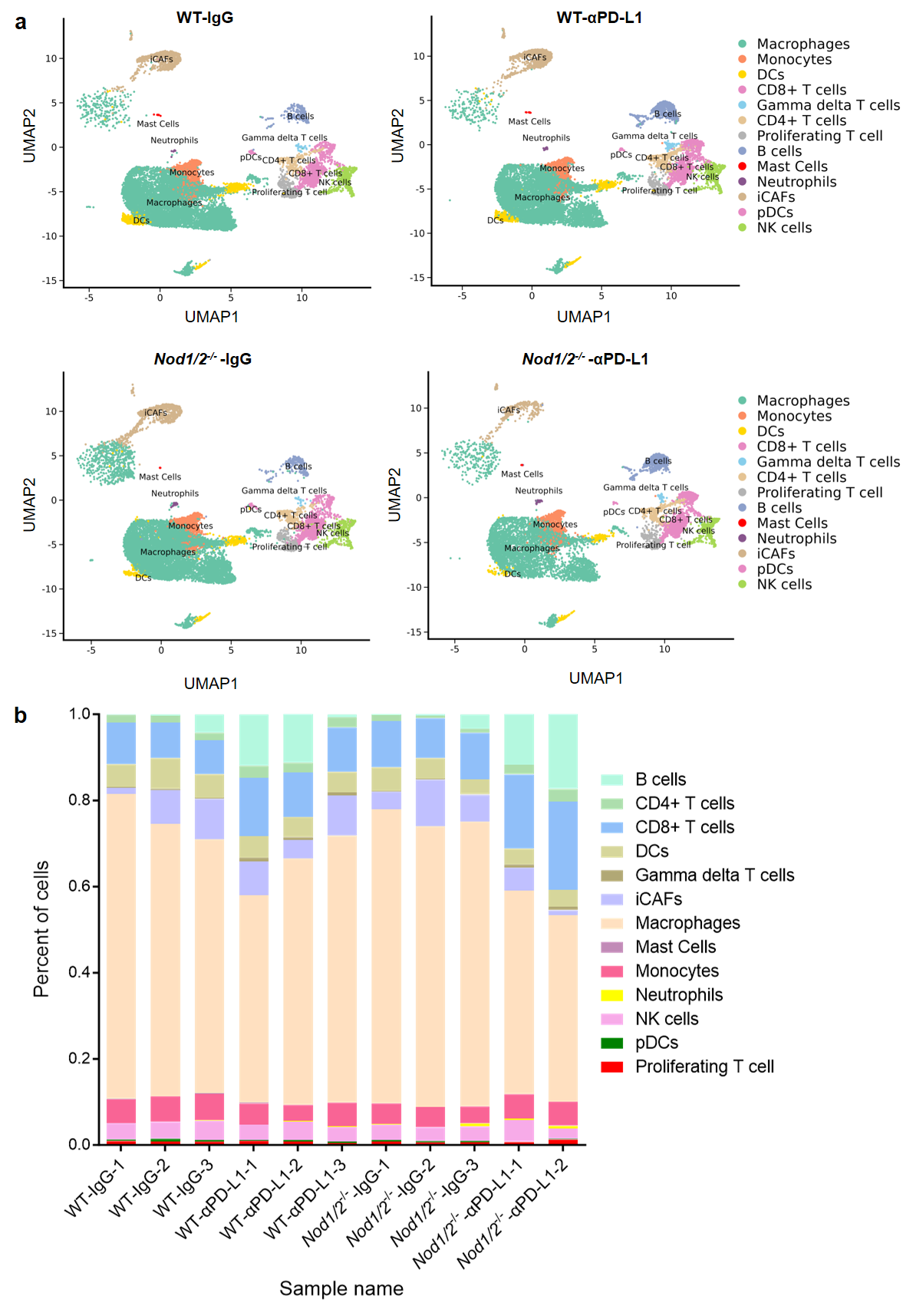


**Figure S3. Deficiency of NOD1/2 alters immune cell subset composition in MC38 tumor following αPD-L1 therapy. a** UMAP plot displaying distinct cell populations of different groups (WT-IgG, WT-αPD-L1, *Nod1/2*^-/-^-IgG, *Nod1/2^-/-^*-αPD-L1) identified by scRNA-seq (*n* = 3, except *Nod1/2*⁻^/^⁻ (αPD-L1), in which *n* = 2). **b** Bar graphs depicting the relative proportions of identified cell subsets in each sample.

**
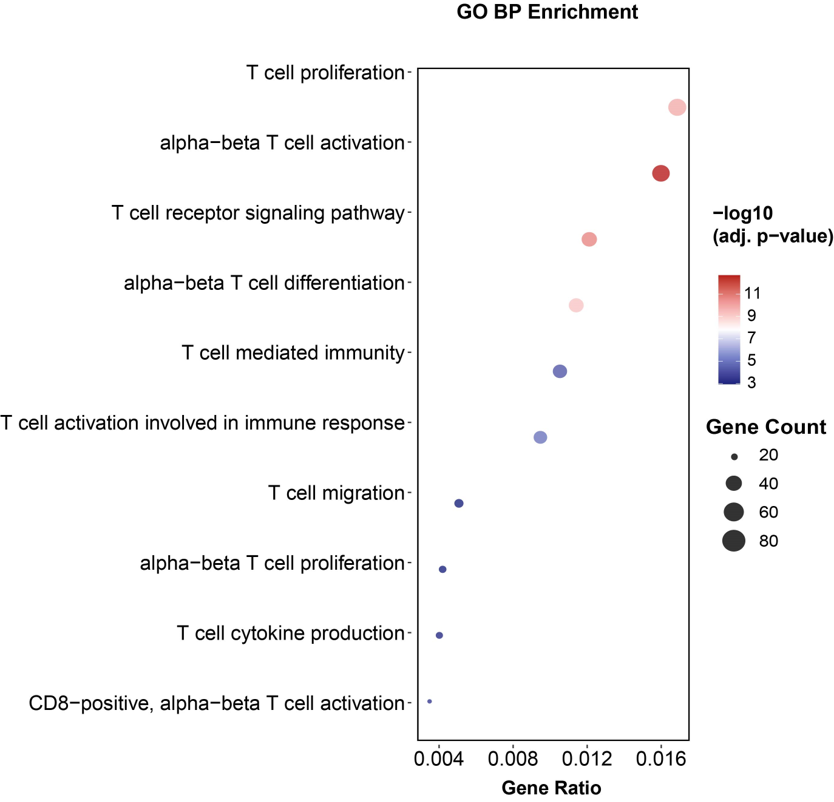
**

**Figure S4. NOD1/2 alters the function of CD8^+^ T cells in MC38 tumor following αPD-L1 therapy.** GO Biological Process (BP) enrichment analysis of differentially expressed genes in CD8^+^ T cells isolated from tumor tissues of WT-αPD-L1 and *Nod1/2^-/-^*-αPD-L1 groups via scRNA-seq.


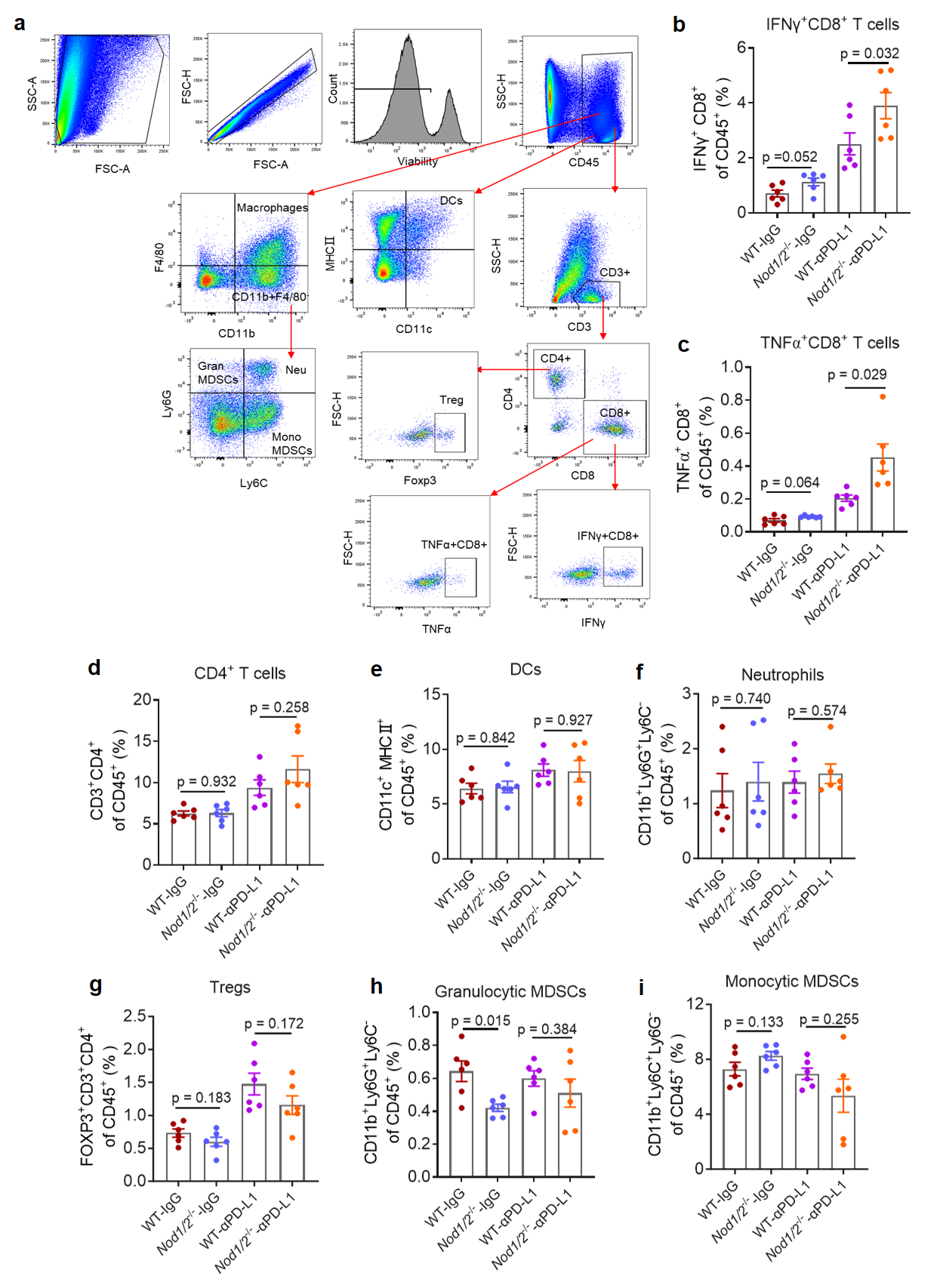


**Figure S5. NOD1/2 signaling impairs tumor immune function following immunotherapy. a** Gating strategy for quantification of immune cell complexity in tumors. **b-i** MC38 tumor bearing mice (WT or *Nod1/2*^-/-^) were treated with IgG or αPD-L1. Tumor-infiltrating immune cell populations were assessed by flow cytometry and quantified as the percentage of CD45^+^ cells (*n* = 6). **b** IFNγ^+^ CD8^+^ T cells; **c** TNFα^+^ CD8^+^ T cells; **d** CD4^+^ T cells; **e** DCs; **f** Neutrophils; **g** Tregs; **h** Monocytic MDSCs; **i** Granulocytic MDSCs. The data are presented as the mean ± SEM. Student’s *t*-test was used to determine the significance level.


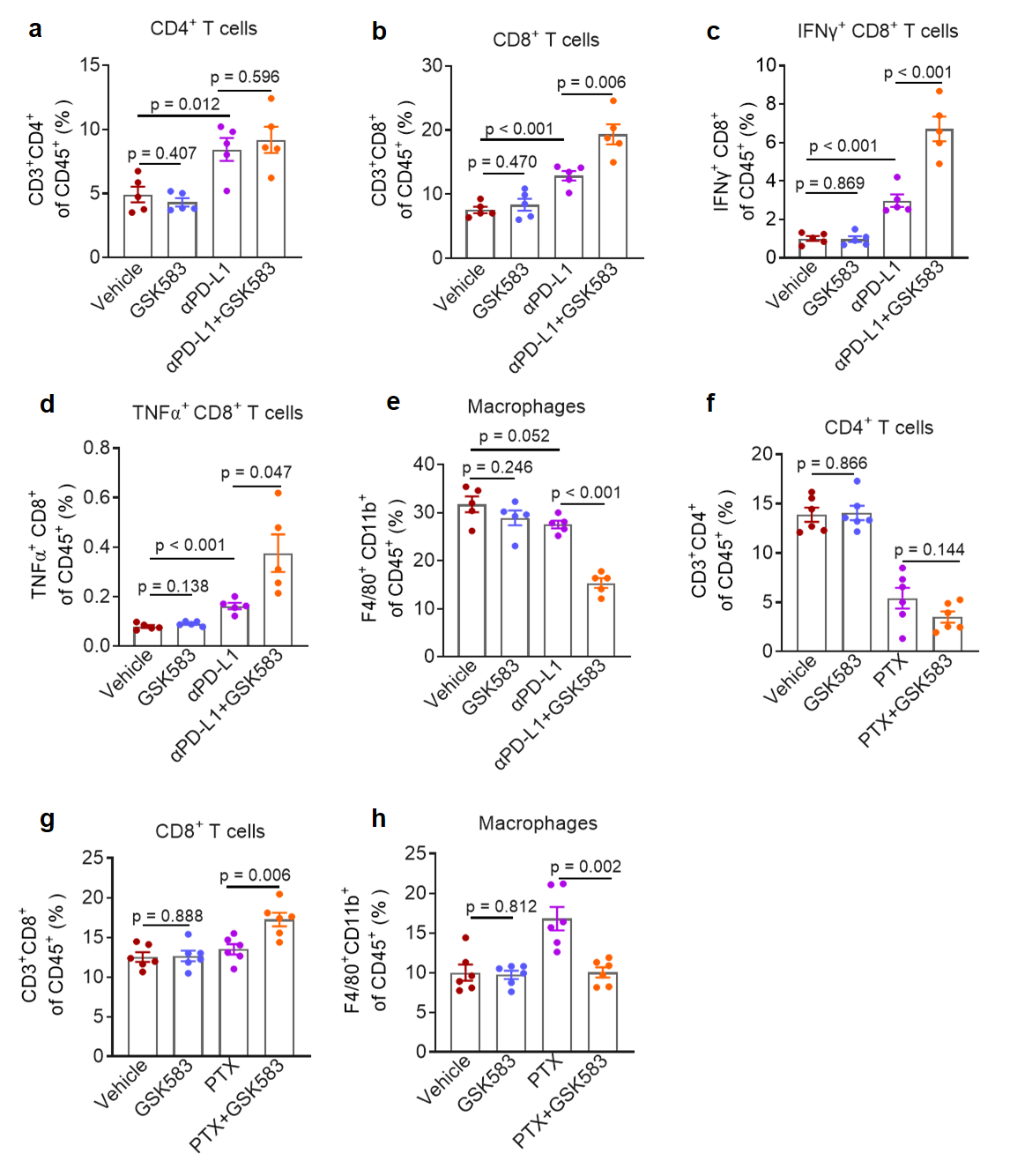


**Figure S6. RIPK2 inhibition remodulates tumor immune function in response to combined immunotherapy and chemotherapy. a-e** MC38 tumor bearing mice were treated with αPD-L1 and/or GSK583. Tumor-infiltrating immune cell populations were analyzed by flow cytometry and expressed as the percentage of CD45^+^ cells (*n* = 5). **a** CD4^+^ T cells; **b** CD8^+^ T cells; **c** IFNγ^+^CD8^+^ T cells; **d** TNFα^+^ CD8^+^ T cells; **e** Macrophages. **f-h** B16F10 tumor bearing mice were treated with PTX and/or GSK583. Tumor-infiltrating immune cell populations were assessed by flow cytometry and quantified as the percentage of CD45^+^ cells (*n* = 6). **f** CD4^+^ T cells; **g** CD8^+^ T cells; **h** Macrophages. The data are presented as the mean ± SEM. Student’s *t*-test was used to determine the significance level.


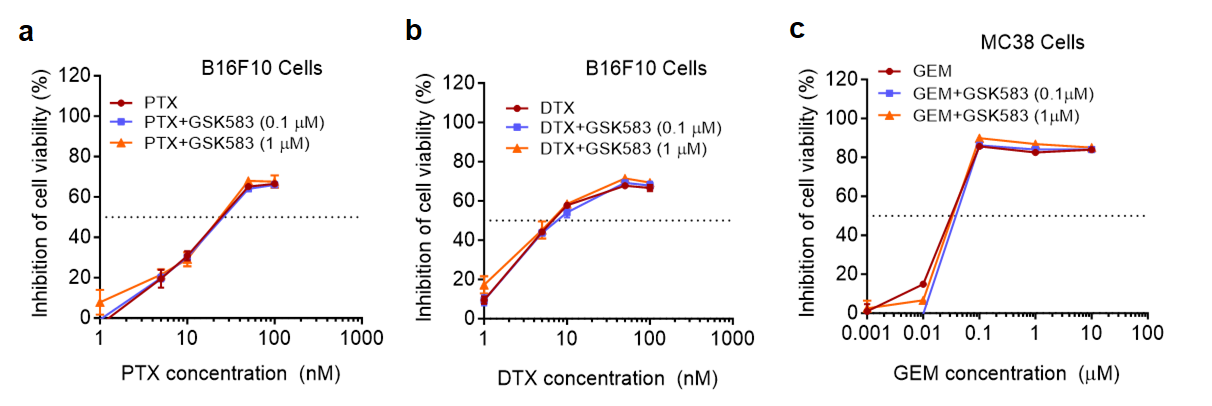


**Figure S7. RIPK2 inhibition does not enhance the *in vitro* cytotoxicity of chemotherapeutic agents. a-c** MTT assay and the corresponding IC_50_ values of PTX, DTX, and GEM with or without GSK583 in tumor cells (*n* = 3). The data are presented as the mean ± SD. Student’s *t*-test was used to determine the significance level.


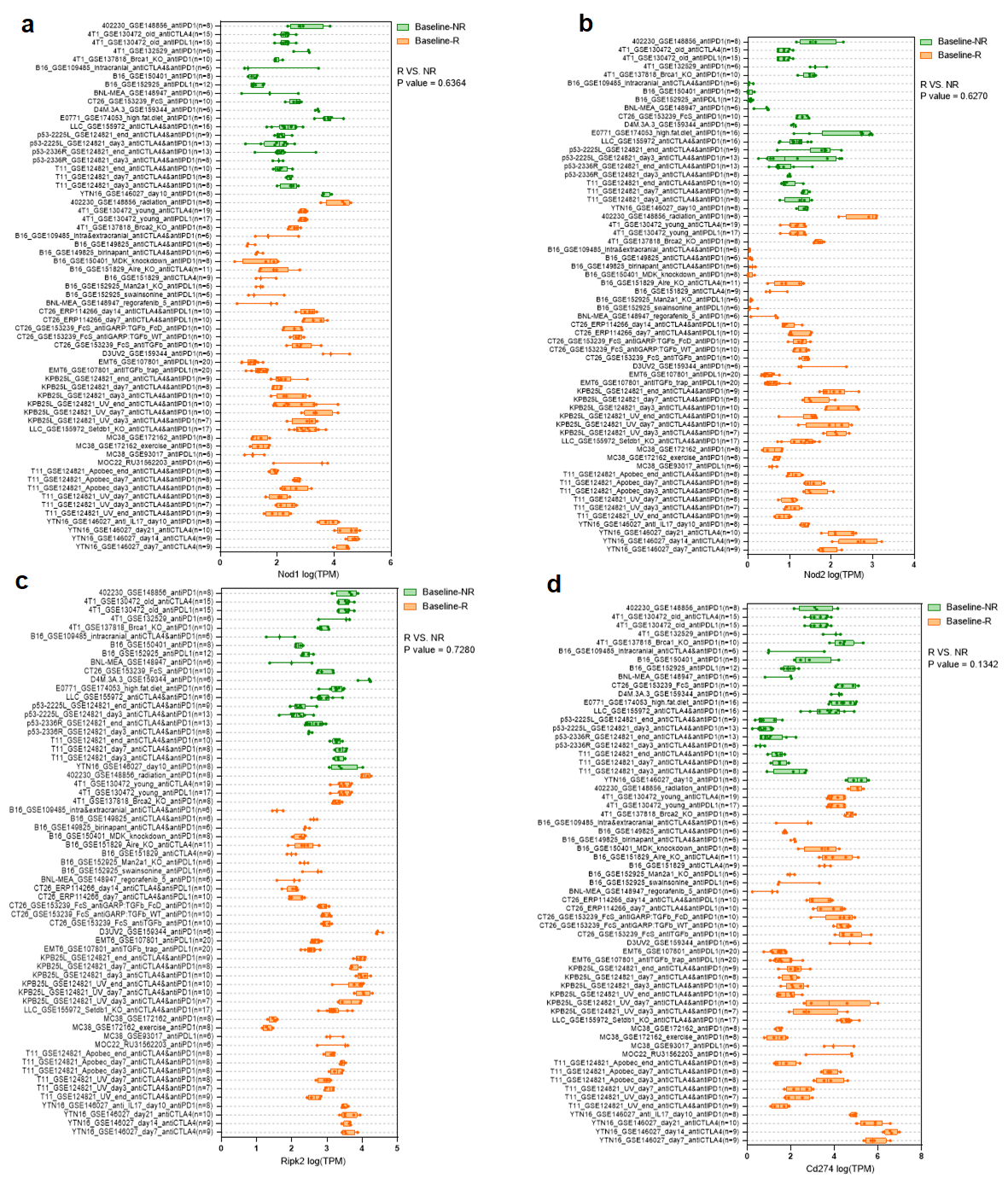


**Figure S8. The expression of NOD1, NOD2, RIPK2 and PD-L1 in tumor tissues across multiple tumor models. a-d** Expression analysis of *Nod1*, *Nod2*, *Ripk2*, and *Cd274* in baseline tumor tissues across multiple tumor models with non-response (NR) or response (R) to immunotherapy. Data were acquired from http://tismo.cistrome.org/. Student’s *t*-test was used to determine the significance level.


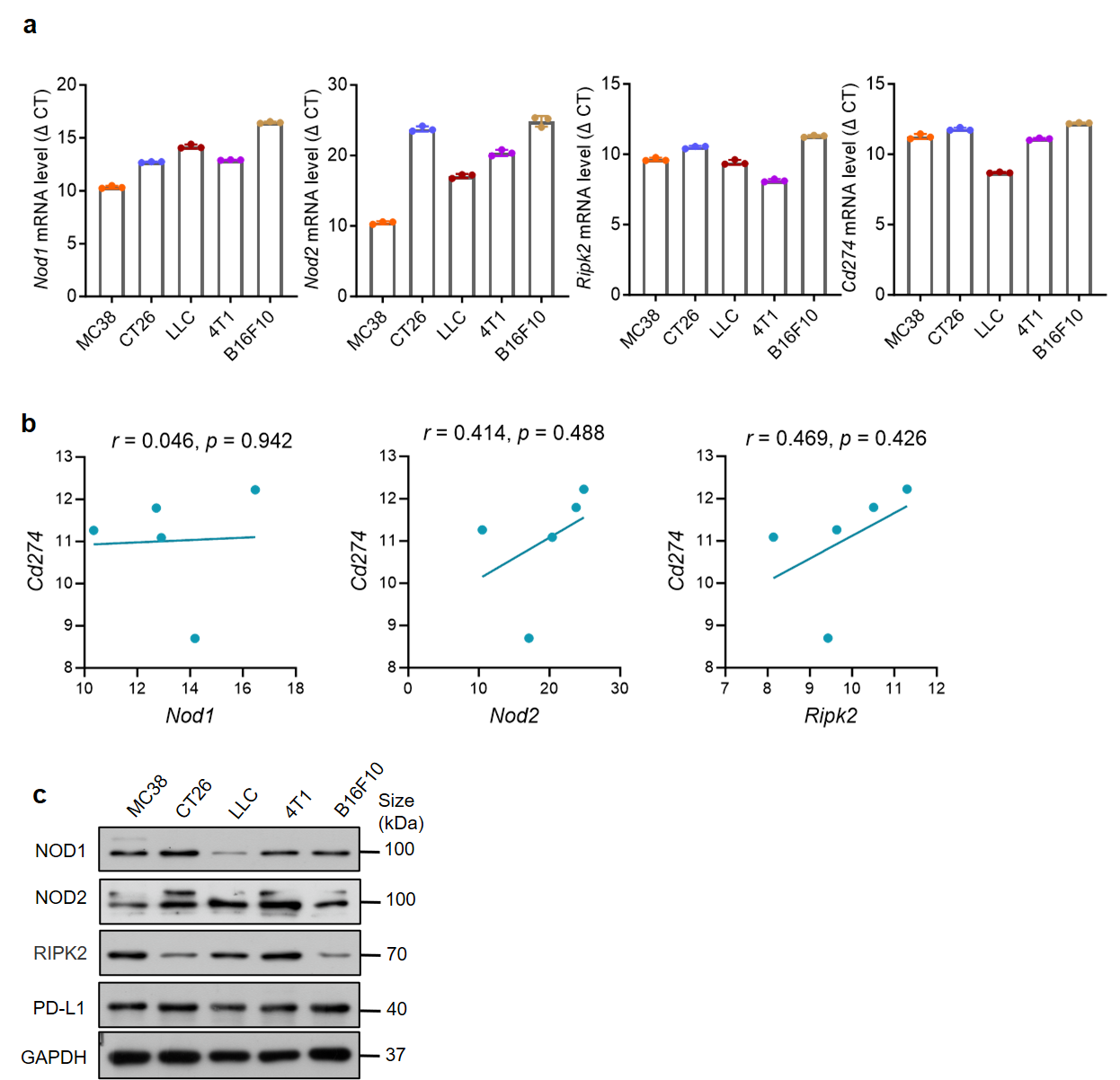


**Figure S9. The correlation between NOD1/2 and PD-L1 in tumor cell lines.** **a** qPCR analyzed mRNA level of *Nod1*, *Nod2*, *Ripk2*, and *Cd274* in different murine tumor cell lines (*n* = 3). **b** Correlation analysis of mRNA level between *Cd274 and Nod1*, *Nod2*, or *Ripk2* in different murine tumor cell lines, assessed using Pearson’s rank correlation. **c** Relative levels of NOD1, NOD2, RIPK2, and PD-L1 in different murine tumor cell lines detected using western blotting. The data are presented as the mean ± SD.


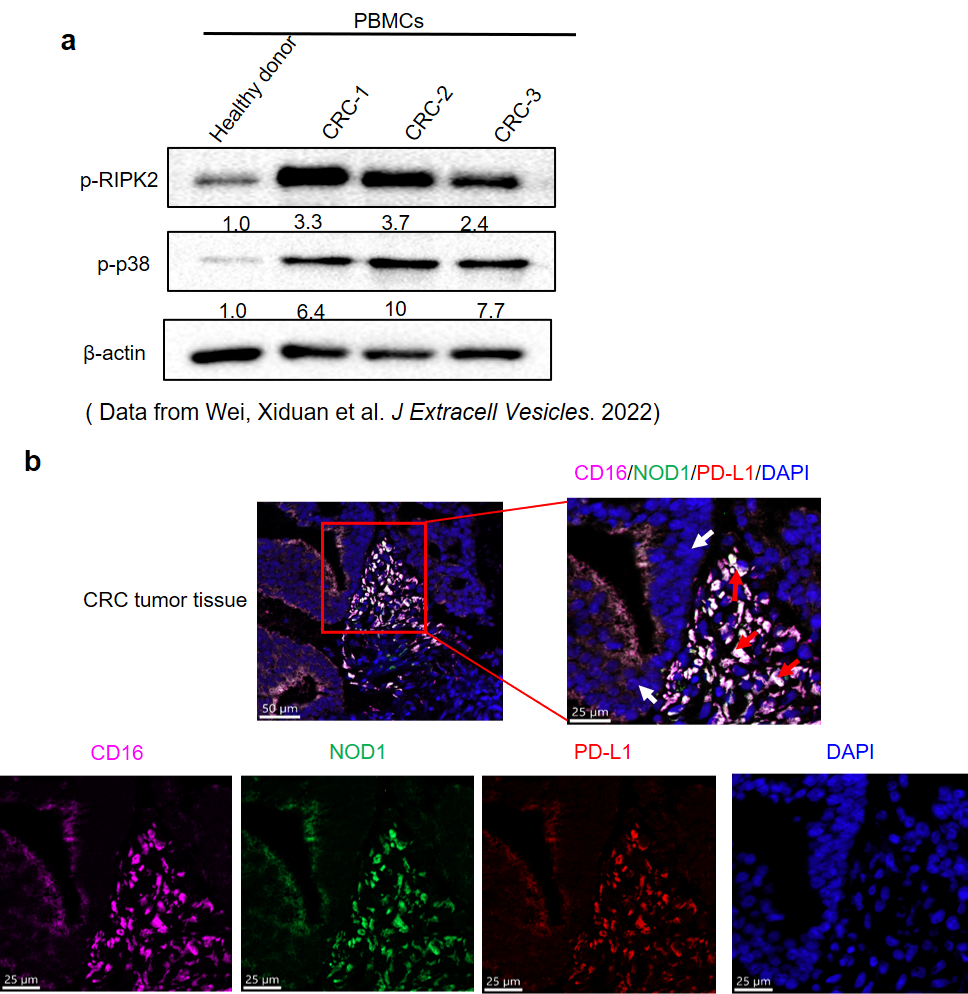


**Figure S10. NOD1 and PD-L1 co-localized in TAMs within MSS-CRC tissues.** **a** Relative level of p-RIPK2 and p-p38 in PBMCs from healthy donors or patients with CRC-LM detected using western blotting and analyzed with ImageJ densitometric software. **b** Representative fluorescent images of NOD1, PD-L1 and macrophage staining in tumor tissues from an MSS-CRC patient. CD16, solferino; NOD1, green; PD-L1, red; DAPI, blue. Red arrow, macrophages; white arrow, tumor tissues. Scale bar, 50 μm and 25 μm.


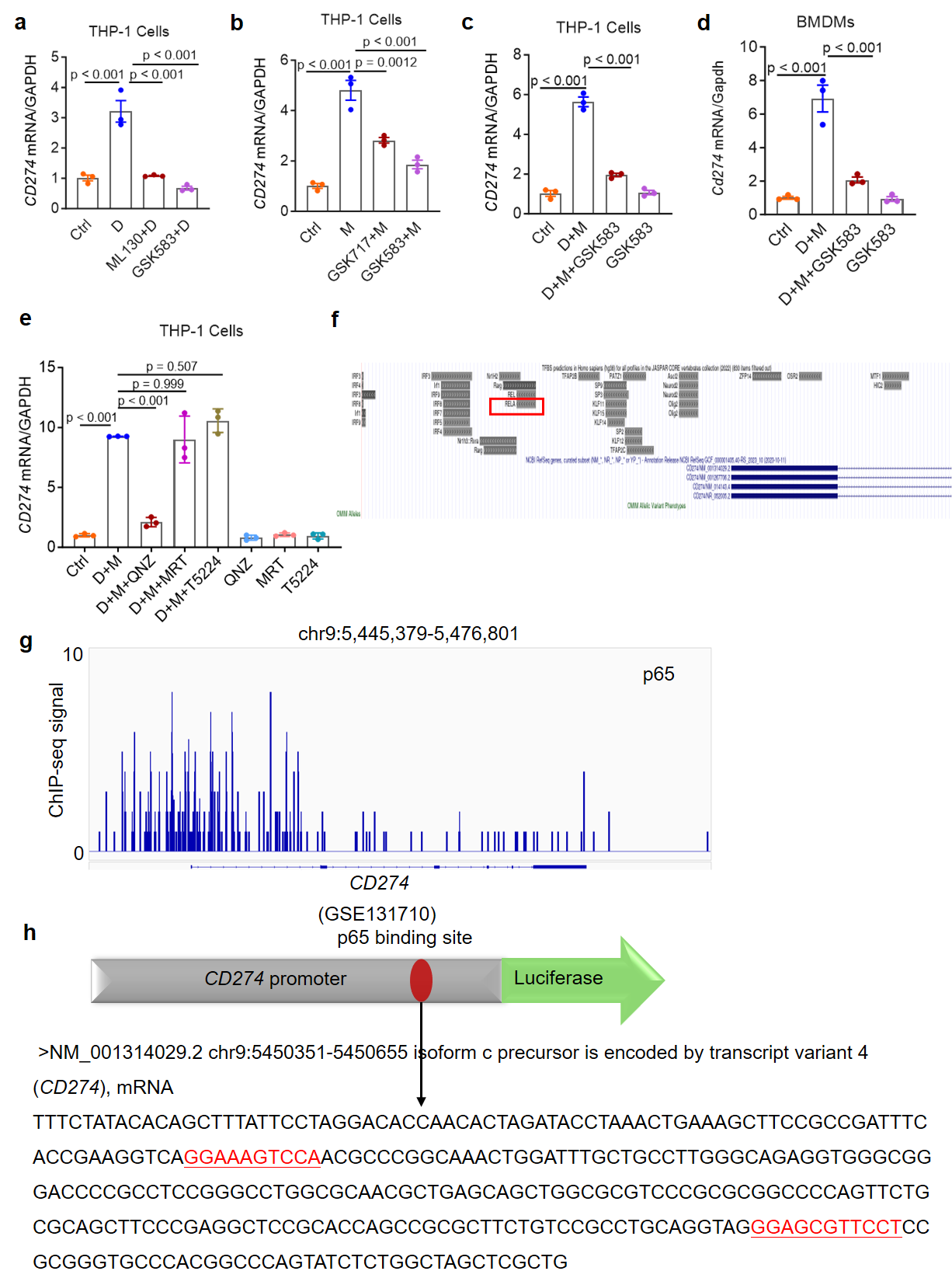


**Figure S11. NOD1/2 regulates PD-L1 expression via the RIPK2/NF-κB pathway. a, b** Relative *CD274* mRNA expression levels in THP-1 cells. **a** Cells were treated with ML130 (NOD1 antagonist, 10 μM) or GSK583 (1 μM) for 1 h before stimulation with C12-iE-DAP (*n* = 3). **b** Cells were treated with GSK717 (NOD2 antagonist, 10 μM) or GSK583 (1 μM) for 1 h before stimulation with MDP (*n* = 3). **c, d** THP-1 and BMDMs were treated with GSK583 (1 μM) for 1 h before stimulation with C12-iE-DAP plus MDP. Relative *Cd274* mRNA expression levels were determined by qPCR (*n* = 3). **e** Relative *CD274* mRNA expression levels in THP-1 cells treated with QNZ (NF-κB inhibitor), T5224 (c-Fos/AP-1 inhibitor), or MRT67307 (IKKε/TBK1 inhibitor) for 1 h before stimulation with C12-iE-DAP and MDP (D+M) (*n* = 3). **f** Analysis of the transcription factors binding to PD-L1 promoter using JASPAR; RELA encodes NF-κB-p65. **g** Analysis of ChIP-seq data from the GEO dataset (GSE131710). **h** Sequence of the *CD274* promoter showing the position of the most representative putative p65 binding sites of the promoter. The data are presented as the mean ± SD. Student’s *t*-test was used to determine the significance level.


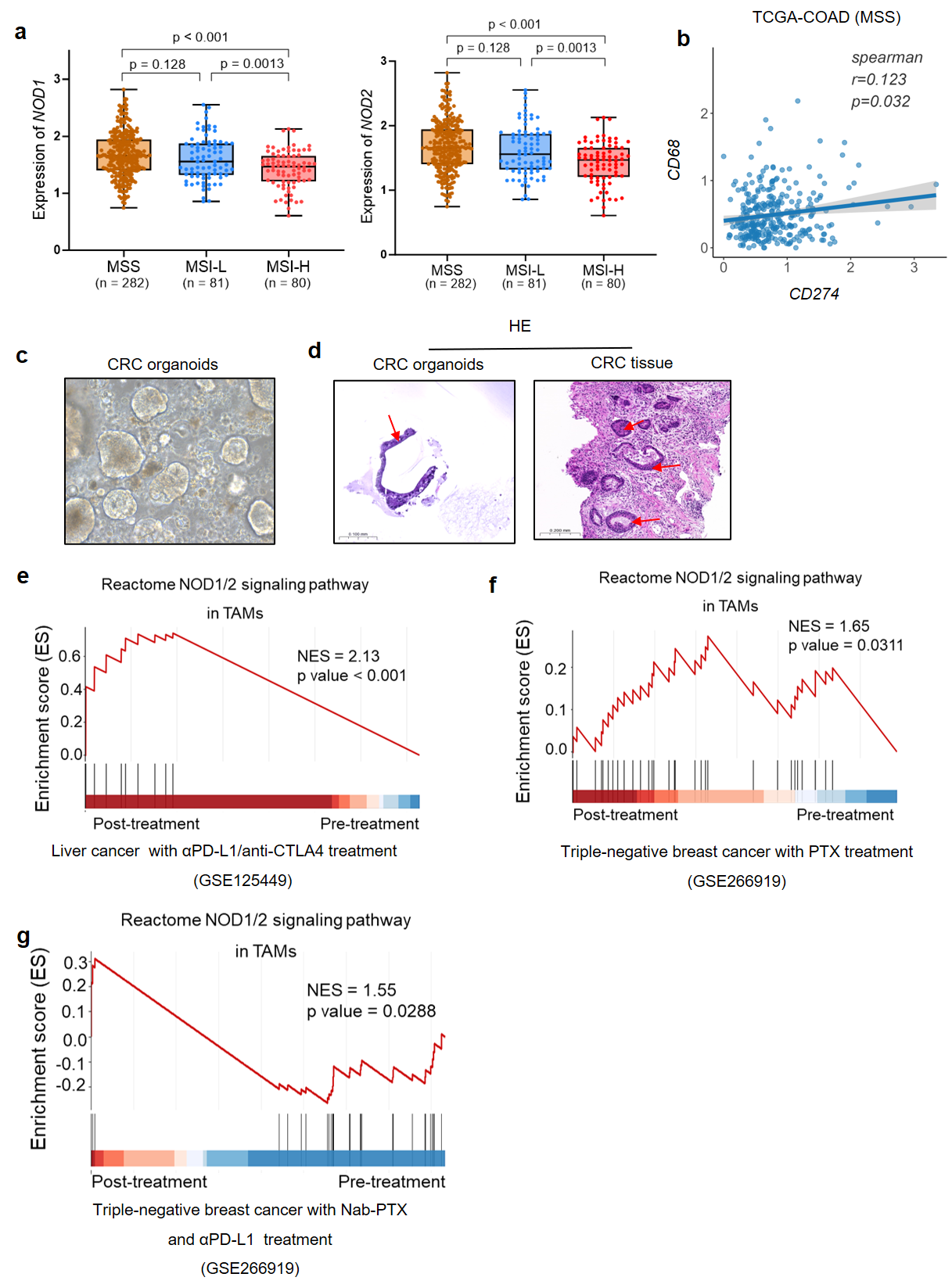


**Figure S12. Analysis of NOD1/2 correlation with PD-L1, organoid model validation, and NOD1/2 signaling in TAMs following various therapies.** **a** Expression analysis of *NOD1* or *NOD2* expression in MSS, MSI-Low (MSI-L), and MSI-High (MSS-H) colon cancer samples from the TCGA cohort. **b** Correlation analysis between *CD274* and *CD68* expression in MSS colon cancer samples from the TCGA cohort (*n* = 282), assessed using Pearson’s rank correlation. **c** Microphotograph images of CRC organoids. **d** HE staining of tumor organoids and original tumor tissue. Organoids show morphology similar to the architecture of the original tumor; red arrow indicates organoids. **e** GSEA of NOD1/2 signaling in TAMs from intrahepatic cholangiocarcinoma tumor samples following PD-L1/CTLA4 antibodies (durvalumab/tremelimumab combination or pembrolizumab alone) treatment compared to the pre-treatment state (*n* = 5, NES = 2.13, *p* = 0.000736). **f** GSEA of NOD1/2 signaling in TAMs from TNBC tumor samples pre- and post-treatment of PTX (*n* = 7, NES = 1.65, *p* = 0.0311). **g** GSEA of NOD1/2 signaling in TAMs from TNBC tumor samples pre- and post-treatment of Nab-PTX+ATZ (*n* = 16, NES = 1.55, *p* = 0.0288).

Supplementary Tables

**Table S1.** Compounds used in the experiments.

| **Commercial Name** | **Compound Structure** | **Biological Function** | **Resource** | **CAS No.**  **(Cat. No.)** |  |
| --- | --- | --- | --- | --- | --- |
| GSK583 |  | RIPK2 inhibitor | MedChemexpress | 1346547-00-9  (HY-100339) | |
| ML130 |  | NOD1 inhibitor | MedChemexpress | 799264-47-4  (HY-18639) | |
| GSK717 |  | NOD2 inhibitor | MedChemexpress | 1595278-21-9  ([HY-136555](https://www.medchemexpress.cn/gsk717.html)) | |
| QNZ |  | NF-κB inhibitor | MedChemexpress | 545380-34-5  (HY-13812) | |
| T5224 |  | AP1 inhibitor | MedChemexpress | 530141-72-1  (HY-12270) | |
| MRT67307 |  | IKKε/TBK1 inhibitor | MedChemexpress | 1190378-57-4  (HY-13018) | |
| Salutaxel  (CBRG001) |  | NOD1/2 inhibitor and tubulin inhibitor | Ningbo Combireg Pharmaceutical Technology Co, Ltd. | NA | |

**Table S2.** Numbers and proportions of immune cell subtypes from scRNA-seq.

| **Cell type**  **Group** | **WT-IgG** | **WT-αPD-L1** | ***Nod1/2^-/-^*-IgG** | ***Nod1/2^-/-^*-αPD-L1** |
| --- | --- | --- | --- | --- |
| B cells | 256 (1.62%) | 1074 (7.71%) | 217 (1.37%) | 1741 (14.75%) |
| CD4^+^ T cells | 260 (1.65%) | 329 (2.36%) | 161 (1.02%) | 308 (2.61%) |
| CD8^+^ T cells | 1360 (8.62%) | 1573 (11.29%) | 1638 (10.33%) | 2246 (19.03%) |
| DCs | 941 (5.96%) | 667 (4.79%) | 714 (4.50%) | 445 (3.77%) |
| Gamm delta T cells | 39 (0.25%) | 100 (0.72%) | 28 (0.18%) | 97 (0.82%) |
| iCAFs | 1015 (6.43%) | 993 (7.13%) | 1103 (6.96%) | 355 (3.01%) |
| Macrophages | 10115 (64.08%) | 7843 (56.29%) | 10543 (66.49%) | 5328 (45.14%) |
| Mast Cells | 16 (0.10%) | 20 (0.14%) | 5 (0.03%) | 5 (0.04%) |
| Monocytes | 928 (5.88%) | 643 (4.62%) | 700 (4.41%) | 657 (5.57%) |
| Neutrophils | 11 (0.07%) | 21 (0.15%) | 56 (0.35%) | 59 (0.50%) |
| NK cells | 637 (4.04%) | 514 (3.69%) | 528 (3.33%) | 426 (3.61%) |
| pDCs | 73 (0.46%) | 52 (0.37%) | 54 (0.34%) | 18 (0.15%) |
| Proliferating T cells | 134 (0.85%) | 103 (0.74%) | 110 (0.69%) | 119 (1.01%) |

**Table S3.** Primer sequences used in qPCR.

| **Gene** | **Primer Sequences** |
| --- | --- |
| Human *CD274* | F: 5’-GCTGCACTAATTGTCTATTGGGA-3’  R: 5’-AATTCGCTTGTAGTCGGCACC-3’ |
| Human *GAPDH* | F: 5’-CGGAGTCAACGGATTTGGTCGTAT-3’  R: 5’-AGCCTTCTCCATGGTGGTGAAGAC-3’ |
| Mouse *Cd274* | F: 5’-GCTCCAAAGGACTTGTACGTG-3’  R: 5’-TGATCTGAAGGGCAGCATTTC-3’ |
| Mouse *Nod1* | F: 5’-GAAGGCACCCCATTGGGTT-3’  R: 5’-AATCTCTGCATCTTCGGCTGA-3’ |
| Mouse *Nod2* | F: 5’-CAGGTCTCCGAGAGGGTACTG-3’  R: 5’-GCTACGGATGAGCCAAATGAAG-3’ |
| Mouse *Ripk2* | F: 5’-ATCCCGTACCACAAGCTCG-3’  R: 5’- GGATGTGTAGGTGCTTCACTG-3’ |
| Mouse *Gapdh* | F: 5’-TGGCCTTCCGTGTTCCTAC-3’  R: 5’-GAGTTGCTGTTGAAGTCGCA-3’ |
